# Quantifying HIV transmission flow between high-prevalence hotspots and surrounding communities: a population-based study in Rakai, Uganda

**DOI:** 10.1016/S2352-3018(19)30378-9

**Published:** 2020-01-14

**Authors:** Oliver Ratmann, Joseph Kagaayi, Matthew Hall, Tanya Golubchick, Godfrey Kigozi, Xiaoyue Xi, Chris Wymant, Gertrude Nakigozi, Lucie Abeler-Dörner, David Bonsall, Astrid Gall, Anne Hoppe, Paul Kellam, Jeremiah Bazaale, Sarah Kalibbala, Oliver Laeyendecker, Justin Lessler, Fred Nalugoda, Larry W Chang, Tulio de Oliveira, Deenan Pillay, Thomas C Quinn, Steven J Reynolds, Simon E F Spencer, Robert Ssekubugu, David Serwadda, Maria J Wawer, Ronald H Gray, Christophe Fraser, M Kate Grabowski, Rakai Health Sciences Program and the Pangea HIV Consortium, Helen Ayles, Rory Bowden, Vincent Calvez, Myron Cohen, Anne Dennis, Max Essex, Sarah Fidler, Dan Frampton, Richard Hayes, Josh Herbeck, Pontiano Kaleebu, Cissy Kityo, Jairam Lingappa, Vladimir Novitsky, Nick Paton, Andrew Rambaut, Janet Seeley, Deogratius Ssemwanga, Frank Tanser, Tom Lutalo, Ronald Galiwango, Fred Makumbi, Nelson K. Sewankambo, Dorean Nabukalu, Anthony Ndyanabo, Joseph Ssekasanvu, Hadijja Nakawooya, Jessica Nakukumba, Grace N. Kigozi, Betty S. Nantume, Nampijja Resty, Jedidah Kambasu, Margaret Nalugemwa, Regina Nakabuye, Lawrence Ssebanobe, Justine Nankinga, Adrian Kayiira, Gorreth Nanfuka, Ruth Ahimbisibwe, Stephen Tomusange, Ronald M. Galiwango, Margaret Nakalanzi, Joseph O. Otobi, Denis Ankunda, Joseph L. Ssembatya, John B. Ssemanda, Emmanuel Kato, Robert Kairania, Alice Kisakye, James Batte, James Ludigo, Abisagi Nampijja, Steven Watya, Kighoma Nehemia, Sr. Margaret Anyokot, Joshua Mwinike, George Kibumba, Paschal Ssebowa, George Mondo, Francis Wasswa, Agnes Nantongo, Rebecca Kakembo, Josephine Galiwango, Geoffrey Ssemango, Andrew D. Redd, John Santelli, Caitlin E. Kennedy, Jennifer Wagman, Aaron Tobian

**Affiliations:** a Department of Mathematics, Imperial College London, London, UK; b Rakai Health Sciences Program, Kalisizo, Old-Bukoba Road, Uganda; c Oxford Big Data Institute, Li Ka Shing Centre for Health Information and Discovery, Nuffield Department of Medicine, University of Oxford, Oxford, UK; d European Molecular Biology Laboratory-European Bioinformatics Institute, Wellcome Genome Campus, Hinxton, UK; e Division of Infection and Immunity, University College London, London, UK; f Department of Medicine, Imperial College London, London, UK; g Department of Medicine, Johns Hopkins School of Medicine, Baltimore, MD, USA; h Department of Pathology, Johns Hopkins School of Medicine, Baltimore, MD, USA; i Division of Intramural Research, National Institute of Allergy and Infectious Diseases, NIH, Bethesda, MD, USA; j Department of Epidemiology, Johns Hopkins Bloomberg School of Public Health, Baltimore, MD, USA; k KwaZulu-Natal Research Innovation and Sequencing Platform College of Health Sciences, University of KwaZulu-Natal, Durban, South Africa; l Department of Statistics, University of Warwick, Coventry, UK; m Makerere University School of Public Health, Kampala, Uganda

## Abstract

**Background:**

International and global organisations advocate targeting interventions to areas of high HIV prevalence (ie, hotspots). To better understand the potential benefits of geo-targeted control, we assessed the extent to which HIV hotspots along Lake Victoria sustain transmission in neighbouring populations in south-central Uganda.

**Methods:**

We did a population-based survey in Rakai, Uganda, using data from the Rakai Community Cohort Study. The study surveyed all individuals aged 15–49 years in four high-prevalence Lake Victoria fishing communities and 36 neighbouring inland communities. Viral RNA was deep sequenced from participants infected with HIV who were antiretroviral therapy-naive during the observation period. Phylogenetic analysis was used to infer partial HIV transmission networks, including direction of transmission. Reconstructed networks were interpreted through data for current residence and migration history. HIV transmission flows within and between high-prevalence and low-prevalence areas were quantified adjusting for incomplete sampling of the population.

**Findings:**

Between Aug 10, 2011, and Jan 30, 2015, data were collected for the Rakai Community Cohort Study. 25 882 individuals participated, including an estimated 75·7% of the lakeside population and 16·2% of the inland population in the Rakai region of Uganda. 5142 participants were HIV-positive (2703 [13·7%] in inland and 2439 [40·1%] in fishing communities). 3878 (75·4%) people who were HIV-positive did not report antiretroviral therapy use, of whom 2652 (68·4%) had virus deep-sequenced at sufficient quality for phylogenetic analysis. 446 transmission networks were reconstructed, including 293 linked pairs with inferred direction of transmission. Adjusting for incomplete sampling, an estimated 5·7% (95% credibility interval 4·4–7·3) of transmissions occurred within lakeside areas, 89·2% (86·0–91·8) within inland areas, 1·3% (0·6–2·6) from lakeside to inland areas, and 3·7% (2·3–5·8) from inland to lakeside areas.

**Interpretation:**

Cross-community HIV transmissions between Lake Victoria hotspots and surrounding inland populations are infrequent and when they occur, virus more commonly flows into rather than out of hotspots. This result suggests that targeted interventions to these hotspots will not alone control the epidemic in inland populations, where most transmissions occur. Thus, geographical targeting of high prevalence areas might not be effective for broader epidemic control depending on underlying epidemic dynamics.

**Funding:**

The Bill & Melinda Gates Foundation, the National Institute of Allergy and Infectious Diseases, the National Institute of Mental Health, the National Institute of Child Health and Development, the Division of Intramural Research of the National Institute for Allergy and Infectious Diseases, the World Bank, the Doris Duke Charitable Foundation, the Johns Hopkins University Center for AIDS Research, and the President's Emergency Plan for AIDS Relief through the Centers for Disease Control and Prevention.

## Introduction

Spatial mapping of infectious diseases, including malaria, tuberculosis, cholera, and HIV has shown considerable spatial heterogeneity in disease prevalence and incidence.^1^, ^2^, ^3^ From a public health perspective, a primary objective of mapping efforts is the identification of so-called hotspots—typically defined as spatial clusters of elevated disease burden or transmission efficiency—to target the highest risk populations, and maximise the public health effect of interventions.^3^ Geographically focused approaches to disease control are supported by modelling studies, which suggest that targeting a small proportion of the population with elevated contact rates and disease incidence (ie, a core group) relative to the overall population has the potential to avert most infections, otherwise known as the 80/20 rule.^4^ However, the overall projected impact of targeted interventions depends on the rate of transmission from core groups to the rest of the population.^1^ Targeting core groups has been used in the control of sexually transmitted infections for decades,^5^ for example gonorrhoea, in which geo-targeted approaches to high-burden areas have proved effective.^6^

**Research in context**

**Evidence before this study**

High-resolution spatial mapping of HIV disease prevalence revealed numerous geographical hotspots of high prevalence throughout the African continent. This information is used to target public health interventions to hyperendemic communities to maximise cost-effectiveness of interventions, and often with the implicit assumption that HIV hotspots serve as sources of transmission to the larger, low prevalence populations. We searched PubMed for all article types published between database inception and April 15, 2019, using search terms related to “HIV”, “hotspots”, “core groups”, “spatial”, and “Africa”. Few studies have investigated the flow of HIV infection between foci of high prevalence (ie, hotspots) and relatively lower prevalence areas using empirical methods. East African communities along Lake Victoria represent some of the highest prevalence communities worldwide with levels greater than three times those in the inland population. One previous study investigated direction of HIV flow between Lake Victoria fishing and inland communities in Uganda using phylogenetic analysis of HIV consensus sequences. However, this study did not identify linkages between inland and fishing communities because of low sampling fractions, and was unable to quantify the flow of infection between the populations. Furthermore, it did not integrate data for population mobility patterns.

**Added value of this study**

In this study, we used HIV deep sequence data from a population-based sample to reconstruct directed HIV transmission networks, and examine the epidemic dynamics between geographical hotspots with high HIV burden along Lake Victoria and surrounding inland communities. We used data from the Rakai Community Cohort Study, an open population-based cohort that provided a high sampling fraction of the communities, which is rare in phylogenetic studies, as well as detailed information on individual-level human migration patterns. We integrated HIV phylogenetic and human migration data, and showed that Lake Victoria and inland epidemics are largely distinct based on a sample of 293 phylogenetically highly supported transmission pairs. Where there is cross-community transmission, it is predominantly from inland to Lake Victoria fishing communities and not vice versa. We also show that men are more likely than women to transmit HIV, and that migrants do not contribute to onward HIV transmission in excess of their prevalence in the population.

**Implications of all the available evidence**

Our findings showed that within sub-Saharan Africa, HIV transmission networks in high prevalence areas can be largely disconnected from those in adjacent lower prevalence populations, and caution against equating and stigmatising HIV hotspots in sub-Saharan Africa universally as population groups that drive disease spread. Geographical targeting of high prevalence areas is essential for local populations in hotspots, but under the same conditions as in the fishing communities of the Rakai Community Cohort Study would have a limited effect on the HIV epidemic in neighbouring lower prevalence communities.

With respect to HIV, the President's Emergency Plan for AIDS Relief, the Global Fund, WHO, and UNAIDS among others have advocated geographical targeting of HIV control interventions to hotspots.^7^, ^8^ These recommendations include calls for HIV elimination in the USA, based on targeting of geographical hotspots to “disrupt the kinetics of HIV spread”.^9^ Although targeting interventions to high-burden populations is ethically justified, and necessary for reducing HIV morbidity and mortality, it is unclear whether such focused approaches would also reduce transmission more broadly. In some cases, HIV hotspots and other high-prevalence groups have been directly or implicitly assumed to constitute core groups disproportionately disseminating infection to the wider transmission network.^10^, ^11^ This assumption, while potentially stigmatising for residents living in hotspots, implies that geographically focused interventions would not only have a direct impact in the targeted geographies but also indirect benefits in the broader population. However, this theory of infection flow from high to low burden populations is rarely confirmed in practice, in part because it is difficult to empirically measure.

In sub-Saharan Africa, where two-thirds of new HIV infections worldwide occur, hotspots include fishing communities bordering the Great Lakes of east and central Africa. These communities typically have a high HIV prevalence, ranging from 20% to 40%, and HIV incidence exceeding 3% annually.^12^, ^13^, ^14^ Historically, Lake Victoria fishing communities also have populations with high levels of mobility, HIV-related risk behaviours, and high sexual contact rates, as well as limited access to health services relative to inland east African populations.^12^, ^13^, ^15^ In 2013, the Ugandan Ministry of Health classified Lake Victoria fishing communities as priority populations for targeted combination HIV prevention services including antiretroviral therapy (ART) at time of HIV diagnosis irrespective of CD4 cell count, HIV counselling and testing, male circumcision, and risk reduction education.^16^ The rationale for targeting fishing communities was based on their high HIV burden, and because they were believed to be acting as core groups sustaining the generalised inland epidemic.^17^

Here, we integrated viral phylogenetic and epidemiological data to empirically measure HIV transmission flows between high HIV prevalence hotspots on Lake Victoria and larger neighbouring inland populations with substantially lower HIV burden in the Rakai region of southern Uganda. We reconstructed directed, partial HIV-1 transmission networks using deep-sequence viral phylogenetic data. Given the high population mobility, networks were interpreted in conjunction with individual-level data for migration patterns to measure transmission flows between fishing and inland communities. We hypothesised that the predominant mode of cross-community infection would be from the high-prevalence hotspots on Lake Victoria to the lower prevalence inland population.

## Methods

### Study design and participants

**Figure 1:**
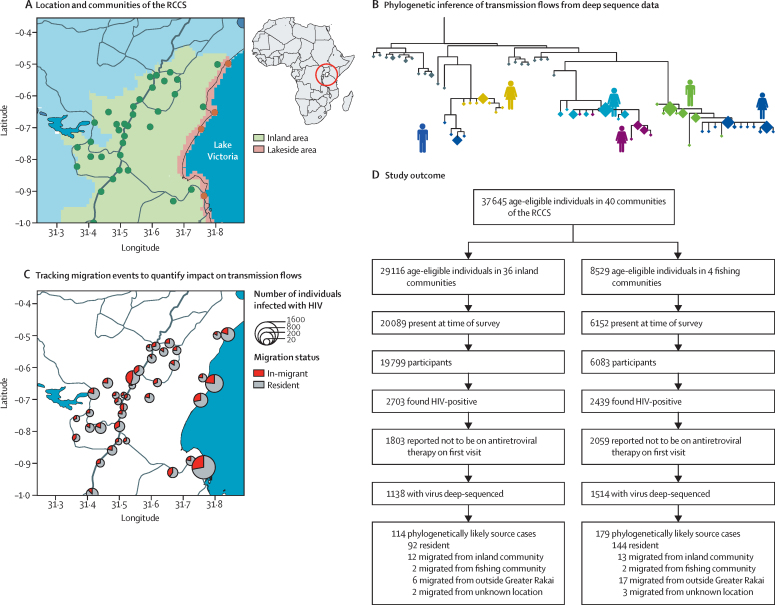
Study design (A) Locations of the RCCS in eastern Africa (left) and the Rakai region of Uganda where the RCCS survey was done (right). The RCCS included an estimated 75·7% of populations in the lakeside area within 3 km of the Lake Victoria shoreline (light brown), and 16·2% of populations in the inland area of the Rakai region (light green). Areas classified as external in this study are shown in light blue. Not shown is one RCCS community northwest outside the map, in which virus sequences were not obtained. (B) The phyloscanner approach for inferring directed HIV transmission networks from deep sequence phylogenies based on ancestral relationships between infecting viruses. With viral deep-sequencing, co-circulating HIV lineages within hosts are represented by many distinct sequence fragments in the data (diamonds, size indicating frequency with which distinct virus was sequenced). In the corresponding phylogenies, sequences from the same individual tend to form subtrees (colours, one for each of the six individuals shown). The ordering of subtrees provides evidence of the direction of transmission. (C) Scale of in-migration into the cohort. For this purpose, RCCS participants were classified as in-migrants if they in-migrated into the cohort in the 2 years before their first visit in the observation period, and otherwise as residents. The panel shows the proportion of in-migrants and residents as well as the size of the population infected with HIV. (D) Key study outcomes including participation, sequencing, and linkage rates. RCCS=Rakai Community Cohort Study.

We did a population-based study using data from the Rakai Community Cohort Study (RCCS) in 36 inland communities of the Rakai region in south-central Uganda shown in figure 1A, and the main four fishing communities within 3 km of Lake Victoria.^13^ The data were collected in two survey rounds in inland communities, and three survey rounds in fishing communities. For each survey round, the RCCS did a household census with GPS coding of household location, followed by a survey of consenting eligible participants aged 15–49 years to ascertain sociodemographic characteristics, sexual risk behaviours, health, and health service use. The RCCS included all age-eligible individuals capable of providing informed consent and resident within the RCCS communities for at least 1 month with the intention to stay. Detailed information was collected on migration history and sexual partners within the past year. HIV status was determined through rapid tests at the time of survey, and confirmatory enzyme immunoassays. All participants were provided with pre-test and post-test counselling, and referral of individuals who were HIV-positive for ART. Individuals were identified using permanent population identifiers, confirmed by photo identification. The last available HIV test result in the observation period was used to establish the infection status of participants (appendix p 8).

The surveyed fishing and inland populations were not proportionate samples of the underlying lakeside population within 3 km of Lake Victoria and the inland region shown in figure 1A. To account for differential sampling, we estimated population sizes in these areas by aggregating high-resolution population density estimates from the WorldPop project, which were checked against population statistics from the Ugandan Bureau of Statistics where possible (appendix p 18).

The study was independently reviewed and approved by the Ugandan Virus Research Institute, Scientific Research and Ethics Committee, protocol GC/127/13/01/16; the Ugandan National Council of Science and Technology; and the Western Institutional Review Board, protocol 200313317. All study participants provided written informed consent at baseline and follow-up visits using institutional review board approved forms.

### Procedures

To infer transmission networks by phylogenetic analysis, viral sequencing was done on plasma blood samples from all individuals who were infected with HIV who self-reported being ART-naive at the time of the survey. This selection criterion was motivated by the fact that self-reported ART use reflected actual ART use with high specificity and sensitivity, and that 90% of individuals who reported ART use had suppressed virus less than 1000 copies per mL of plasma,^18^ below which viral deep sequencing was not possible within our protocol.^19^, ^20^ Deep sequencing based on the protocol of Gall and colleagues^19^ generated many sequence fragments that capture viral diversity within individuals (figure 1B), which unlike Sanger sequencing, enables phylogenetic inference into the direction of transmission from sequence data alone.^21^ Phylogenetic transmission networks in the population-based sample were reconstructed from deep-sequence data with phyloscanner,^20^, ^22^ which addresses caveats in deep-sequence phylogenetic analysis such as read contamination, and has an estimated false discovery rate in inferring the incorrect direction of transmission of 15–20%.^20^ To capture uncertainty in phylogenetic inferences, phylogenetic relationships were evaluated in a large number of deep-sequence phylogenies sliding across the HIV genome. Pairs of individuals with evidence for phylogenetic linkage and transmission in one direction in at least 60% of phylogeny evaluations were identified and considered to be highly supported source–recipient pairs. The threshold was determined in analysis of pairs with epidemiological evidence on the direction of transmission.^20^

To interpret reconstructed source–recipient pairs in the context of population mobility, migrants were identified at census and defined as people who had moved into a community regardless of distance travelled or whether or not the source community was under RCCS surveillance (figure 1C). To be included in the survey, in-migrating individuals were required to have stayed in the community for at least 1 month or, if this was not the case, they intend to stay in the community for 6 months or longer. In-migrating individuals were classified as such if they in-migrated into the cohort community within 2 years before their first visit in the observation period, and otherwise they were classified as residents. To estimate transmission flows, phylogenetically likely sources were classified as in-migrants if they in-migrated into the cohort in the 2 years before the date at which their transmission recipient was found to be infected, and it was assumed that the individual acquired infection at the community of origin. The community of origin of migration was recorded as a free response variable and geo-coded using Google Earth by Ugandan co-investigators with local expertise. Sensitivity analyses using alternative definitions of in-migrants are described in the appendix (p 22).

### Statistical analysis

Individual geo-location and migration data were used to attribute source and destination locations for each source–recipient pair. The geo-location of each recipient in a pair was set to the community in which the recipient was found to be infected. For the phylogenetically likely source partner, the location was set to the community of residence at or shortly before the recipient was identified as HIV-positive. If the source partner had migrated within the past 2 years, the location was set as the community before migration. To estimate transmission flows in the cohort, we first counted the proportion of source–recipient pairs by source and destination locations (unadjusted estimates). Next, we adjusted for differential RCCS participation and sequence sampling rates by individual-level characteristics with Bayesian multilevel models as detailed in the appendix (p 12) using the phyloflows R package version 1.1.0. Finally, we predicted transmissions flows between inland and fishing areas (predictions). To do this, we scaled the adjusted estimates of transmission flows between RCCS communities by the number of men and women in inland and fishing areas as detailed in the appendix (p 18). Sensitivity analyses are reported in the appendix (p 21). When not specified, adjusted estimates of transmission flows are reported throughout.

### Role of the funding source

The funders of the study had no role in the data collection, data analysis, data interpretation, or writing of the report. The corresponding authors had full access to all the data in the study, and had final responsibility for the decision to submit for publication.

## Results

**Figure 2:**
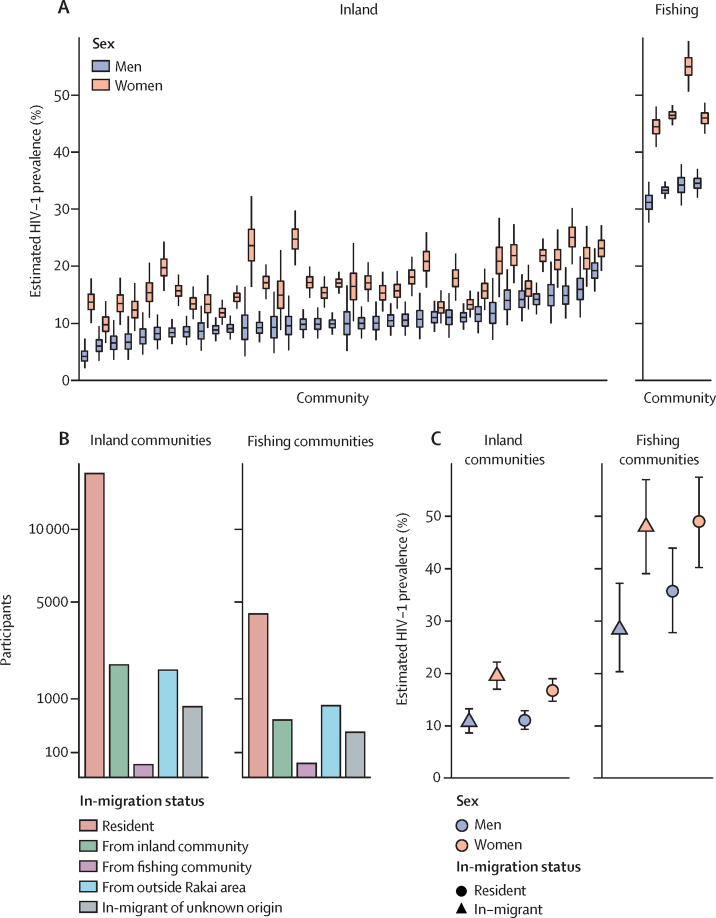
HIV prevalence and migration in inland and fishing communities (A) Estimates of HIV prevalence in RCCS communities for men (blue) and women (pink) in inland communities (left panel) and fishing communities (right panel). Boxplots indicate central estimates (black bar), IQRs (box), and 95% credibility intervals (whiskers). HIV prevalence was substantially higher in fishing communities for both men and women. (B) Number of RCCS participants in inland and fishing communities by in-migration status. Participants who in-migrated within 2 years before study visit were stratified by the origin of migration, from inland communities (green), from fishing communities (purple), from outside the Rakai area (light blue), and from unknown location (grey). (C) Estimates of HIV prevalence among in-migrants to inland communities to that among in-migrants to fishing communities. HIV prevalence was higher among those individuals migrating to fishing communities than those migrating to inland communities. Sex specific estimates in panels A and C were obtained with Bayesian logistic regression models using the Stan software, version 2.19.

From Aug 10, 2011, to Jan 30, 2015, 25 882 individuals in 40 communities participated in the RCCS (figure 1D). An estimated 179 982 individuals aged 15–49 years lived in the inland area. 29 116 (16·2%) were census-eligible residents in the 36 inland RCCS communities, of whom 19 799 (68·0%) participated in the survey and provided a blood sample for HIV detection. Of an estimated 11 272 individuals aged 15–49 years in the lakeside area, 8529 (75·7%) individuals were census-eligible in the four fishing communities of the RCCS, of whom 6083 (71·3%) participated in the survey. Participation rates varied by sex, age, and migration status (appendix p 15). The most common reason for non-participation was absence for work or school (96·9%). In the inland communities, 2703 (13·7%) participants were HIV-positive. In the fishing communities, 2439 (40·1%) participants were HIV-positive. HIV prevalence was higher in the fishing communities than inland communities for both men and women (figure 2A).

6511 (25·2%) of 25 882 survey participants were classified as in-migrants,^23^ of whom 2710 (41·6%) originated from outside the Rakai region, 59 (0·9%) from locations on the shores of Lake Victoria, 2598 (39·9%) from inland locations in the Rakai region, and 1144 (17·6%) had no resolved location of migration origin (figure 2B). In the four RCCS fishing communities, 837 (48·2%) in-migrants originated from outside the Rakai area, 32 (1·8%) from locations on the shores of Lake Victoria, 536 (30·9%) from inland locations in the Rakai area, and 331 (19·1%) had no resolved source location. In the RCCS inland communities, 1873 (39·2%) in-migrants originated from outside the Rakai area, 27 (0·6%) from locations on the shores of Lake Victoria, 2062 (43·2%) from inland locations in the Rakai area, and 813 (17·0%) had no resolved source location. The proportion of in-migrants among study participants was similar in inland and fishing communities (24·1% *vs* 28·5%), but substantially more participants migrated from inland to fishing communities (n=536) than vice versa (n=27). Among the in-migrants who moved into fishing communities, HIV prevalence was significantly higher than among in-migrants who moved into inland communities (figure 2C).

There were 3878 (75·4%) individuals infected with HIV who reported no ART use on at least one survey visit in the observation period, and the first sample from these individuals was prepared for viral sequencing (appendix p 5). Deep-sequencing yielded output of moderate quality.^24^ Analysis was restricted to samples from 2652 individuals who satisfied minimum criteria on read length and read depth,^20^ which implied a sampling fraction of 68·4% among participants who were infected and self-reported being ART-naive, and an estimated sampling fraction of 45·1% among census-eligible individuals who were infected with unsuppressed virus.^20^ Sequence sampling rates varied by sex, age, migration status, and across RCCS communities (appendix p 16).

From the population-based deep-sequencing data, 446 HIV transmission networks were reconstructed, which included 293 source–recipient pairs with strong phylogenetic support for epidemiological linkage and the direction of transmission (appendix p 5). Following previous arguments,^20^ we expected that for approximately 800 (30·2%) of the 2652 sampled individuals, our data also contain sequences from their transmitter, suggesting that phylogenetic analysis probably did not identify all transmission events between sampled individuals. In 57 (19·5%) of the source–recipient pairs, the likely transmitter had migrated in the 2 years before diagnosis of the recipient, suggesting that the current residence of the likely transmitter at the time of the survey was not necessarily the location at which they acquired infection. We defined the source location for these 57 pairs as the origin of migration of the likely transmitter. Sensitivity analyses that re-defined the source location for likely transmitters who in-migrated in 6, 12, 36, and 48 months before diagnosis of the recipient are reported in the appendix (p 22), and did not substantially change our results.

**Figure 3:**
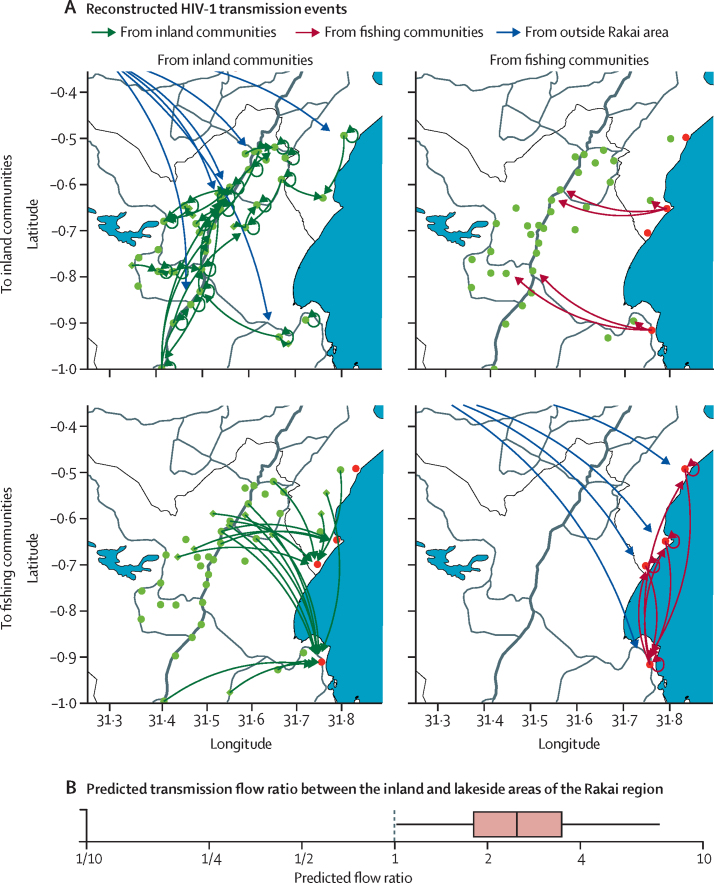
Phylogenetically highly supported transmission flows in the population-based sample, and predicted transmission flows Viral deep-sequence phylogenetics identified 293 source–recipient pairs with strong phylogenetic support for epidemiological linkage and the direction of transmission. Transmission events were geo-located to the communities in which the phylogenetically likely sources and recipients had their households, or to the origin of recent in-migration events. (A) Phylogenetically reconstructed transmission events. 94 phylogenetically reconstructed transmissions events occurred from inland to inland communities, and six occurred from outside the Rakai area to inland communities; seven were observed from fishing to inland communities; 23 occurred from inland to fishing communities; 141 occurred from fishing to fishing communities, and 17 from outside the Rakai area to fishing communities. Not shown are two phylogenetically probable transmission events with unknown source location to inland communities, and three such events to fishing communities. (B) Predicted transmission flow ratio among populations living in inland and lakeside areas of the Rakai region, after adjusting for survey, participation, and sequence sampling bias. The predicted flow ratio of transmissions from inland to lakeside areas compared with the opposite direction was 2·50 (95% CrI 1·02–7·30).

Figure 3 shows the transmission flows inferred among the 293 source–recipient pairs. 235 (80·2%) transmission events occurred within inland communities or within fishing communities, 30 (10·4%) occurred between them, 23 (7·8%) were from outside the Rakai region, and five (1·7%) had an unknown source location (table). There were more transmissions from inland to fishing communities than vice versa (23 *vs* seven). The unadjusted flow ratio from inland to fishing communities was 3·29 (23 of 264 to seven of 264).

**Table:** HIV-1 transmission between sites with high and low HIV-1 prevalence in Greater Rakai, Uganda

	**Recipient population**	Phylogenetically supported transmission flows among RCCS communities*	**Estimated contribution to overall HIV-1 transmission among RCCS communities**†**(posterior mean)**	**Predicted contribution to overall HIV-1 transmission among inland and lakeside areas of the Rakai region**‡**(posterior predictive mean)**
**Source overall**				
Fishing communities	Fishing communities	141 (48·1%)	44·5% (38·6–50·5)	5·2% (3·8–7·0)
Fishing communities	Inland communities	7 (2·4%)	3·5% (1·7–6·3)	1·7% (0·6–3·5)
Inland communities	Fishing communities	23 (7·8%)	7·8% (5·1–11·3)	4·3% (2·3–7·0)
Inland communities	Inland communities	94 (32·1%)	35·5% (29·8–41·6)	88·7% (84·5–91·9)
External to Rakai area	Fishing communities	17 (5·8%)	5·8% (3·5–8·9)	..
External to Rakai area	Inland communities	6 (2·0%)	2·5% (1·0–5·0)	..
Unknown origin	Fishing communities	3 (1·0%)	..	..
Unknown origin	Inland communities	2 (0·7%)	..	..
**Source by sex**				
Men, fishing communities	Women, fishing communities	79 (27%)	25·3% (20·6–30·5)	2·9% (2·0–4·2)
Men, fishing communities	Women, inland communities	6 (2·0%)	3·1% (1·4–5·9)	1·5% (0·5–3·2)
Men, inland communities	Women, fishing communities	12 (4·1%)	4·2% (2·2–7·0)	2·5% (1·1–4·8)
Men, inland communities	Women, inland communities	59 (20·1%)	22·3% (17·4–27·7)	55·8% (44·5–66·2)
Men, external to Rakai area	Women, fishing communities	8 (2·7%)	2·6% (1·2–4·9)	..
Men, external to Rakai area	Women, inland communities	4 (1·4%)	1·5% (0·5–3·7)	..
Men, unknown origin	Women, fishing communities	3 (1·0%)	..	..
Men, unknown origin	Women, inland communities	2 (0·7%)	..	..
Women, fishing communities	Men, fishing communities	62 (21·2%)	19·0% (14·8–23·8)	2·2% (1·5–3·2)
Women, fishing communities	Men, inland communities	1 (0·3%)	0·2% (0·0–1·2)	0·1% (0·0–0·9)
Women, inland communities	Men, fishing communities	11 (3·8%)	3·5% (1·8–6·0)	1·7% (0·7–3·3)
Women, inland communities	Men, inland communities	35 (11·9%)	13·1% (9·4–17·6)	32·8% (22·7–44·0)
Women, external to Rakai area	Men, fishing communities	9 (3·1%)	3·0% (1·5–5·5)	..
Women, external to Rakai area	Men, inland communities	2 (0·7%)	0·8% (0·1–2·5)	..
Women, unknown origin	Men, fishing communities	0	..	..
Women, unknown origin	Men, inland communities	0	..	..

Data are n (%) or mean (95% credibility interval). RCCS=Rakai Community Cohort Study.

* Phylogenetically reconstructed transmission events, unadjusted.

† Estimates based on phylogenetically reconstructed events, and adjusted for participation and sequencing differences via a Bayesian multi-level model; see appendix p 11.

‡ Predictions based on a fitted Bayesian multi-level model, and extrapolated from eligible individuals who live in RCCS communities to the inland and fishing areas shown in figure 1A; see appendix p 18.

After adjusting for variation in participation and sequencing rates, an estimated 80·1% (95% credibility interval [Crl] 75·1–84·6) of transmissions were within inland communities or within fishing communities, 7·8% (5·1–11·3) were from inland to fishing communities, 3·5% (1·7–6·3) were from fishing to inland communities, 5·8% (3·5–8·9) were from outside the Rakai region to fishing communities, and 2·5% (1·0–5·0) were from outside the Rakai region to inland communities (table). Considering that the study population comprised an estimated 16·2% of the inland population and 75·7% of the lakeside population of the region where the RCCS survey was done, it is only possible to interpret the combined estimate of transmission flows within inland and within fishing communities. The estimated adjusted flow ratio from inland to fishing communities was 2·25 (95% CrI 1·04–5·23).

We next scaled the adjusted estimates within and between RCCS communities to the populations living in inland and lakeside areas of the Rakai region shown in figure 1A, giving a prediction for transmission patterns within the inland and the lakeside areas in which the study communities are located. The predicted proportion of transmissions was 88·7% (95% CrI 84·5–91·9) within the inland area, 5·2% (3·8–7·0) within the lakeside area, 4·3% (2·3–7·0) from inland to lakeside areas, and 1·7% (0·6–3·5) from lakeside to inland areas (table). The predicted flow ratio from inland to lakeside areas was 2·5 (95% CrI 1·0–7·3). We report estimates of the sources of infection in each of the population groups and the recipients of transmissions from each of the population groups in the appendix (p 6).

**Figure 4:**
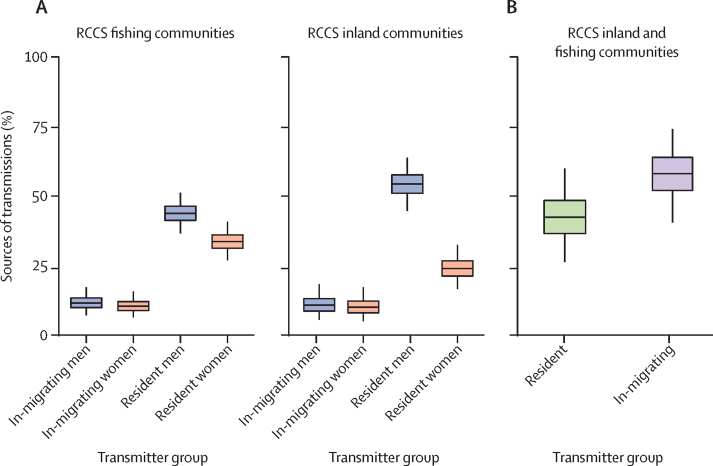
Effect of sex and migration on transmission flows (A) Estimated sources of transmission in inland and fishing communities of the RCCS. (B) Estimated amount of cross-community transmissions between inland and fishing communities originating from residents with partners outside their community and from in-migrants. Estimates in both panels were obtained as described in the appendix (p 11), and adjusted for heterogeneity in participation and sequence sampling. In fishing communities, an estimated 33·6% (95% CrI 26·7–40·7) of transmissions originated from resident women, 43·7% (36·7–51·1) from resident men, 10·5% (6·5–15·8) from in-migrating women, and 11·7% (6·5–15·8) from in-migrating men. In inland communities, an estimated 24·0% (95% CrI 16·7–33·5) of transmissions originated from resident women, 54·2% (44·7–63·6) from resident men, 10·1% (5·1–17·3) from in-migrating women, and 10·9% (5·6–18·4) from in-migrating men. Boxes are 50% CrI and whiskers are 95% CrI. RCCS=Rakai Community Cohort Study. 95% CrI=95% credible interval.

An estimated 59·7% (95% CrI 53·9–65·3) of transmissions in RCCS communities originated from men, whereas the majority of infected participants were women (3149 [61·2%] of 5142). To investigate this transmission bias, we stratified the source populations in inland and fishing communities by sex and migration status (figure 4A). The gender bias was larger in inland communities than fishing communities (appendix p 3). Further, in inland communities, the ratio in transmissions from in-migrating men compared with in-migrating women was 1·07 (95% CrI 0·44–2·66), and the ratio of transmissions from resident men compared with resident women was 2·26 (1·45–3·62). In fishing communities, the ratio in transmissions from in-migrating men compared with in-migrating women was 1·11 (95% CrI 0·57–2·16), and the ratio of transmissions from resident men compared with resident women was 1·30 (0·94–1·82).

An estimated 22·1% (95% CrI 17·3–27·5) of all transmissions originated from in-migrants, which was not larger than the proportion of participants who were infected who were in-migrants (appendix p 4). We further suspected that migration of individuals who were HIV-positive could have a larger effect specifically on cross-community transmissions between fishing and inland communities, and thus we quantified the proportion of cross-community transmissions that resulted from migration (figure 4B). An estimated 42·2% (95% CrI 26·1–59·7) of cross-community transmissions originated from residents of inland communities who had partners in fishing communities (or vice versa from residents of fishing communities who had partners in inland communities), and 57·8% (95% CrI 40·3–73·9) originated from in-migrants from inland communities to fishing communities who had partners in fishing communities (or vice versa).

## Discussion

Understanding the extent to which geographical areas with a high HIV burden contribute to transmission in neighbouring lower prevalence populations is important for guiding targeted HIV control efforts. In this study, we reconstructed HIV transmission networks in southern Uganda using deep-sequence HIV phylogenetics. We then integrated these data with individual-level information on migration to assess whether high HIV burden fishing communities (around 40% prevalence) along Lake Victoria are major sources of HIV transmission to larger, lower prevalence inland communities (around 14% prevalence). Previously, it has been shown that Lake Victoria fishing communities are geographical hotspots of HIV prevalence and incidence in the east Africa region.^12^, ^13^, ^14^ However, our results showed that HIV acquisition in the southern Ugandan inland is largely unrelated to the Lake Victoria epidemic. Furthermore, among the few infections between lakeside and inland areas, transmission flow is more than twice as likely to be from inland to fishing areas than vice versa. These results add to earlier findings from smaller Ugandan studies suggesting that the epidemic on the Lake Victoria shores is distinct from that in the east African inland.^25^ Our results, along with previous research, imply that targeted control in these lakeside hotspots, while essential for the local population, would have minimal effect on the epidemic in the larger inland populations, which we found accounts for the vast majority of total transmissions (around 90%).

Transmission flows between high and low prevalence populations have previously been assessed through phylogenetic analyses of consensus genomes.^26^ However, these approaches require modelling assumptions to infer the directionality of transmission between populations. By contrast, transmission flows can be estimated directly from analyses of deep-sequence data that account for within-host HIV diversity and ancestral relationships between viruses.^21^, ^22^ We have previously validated deep-sequence phylogenetic analyses in the Rakai population and showed that although our methods cannot prove direct transmission between two individuals, the direction of transmission can be inferred with sufficient accuracy for epidemiological analysis.^20^ In this analytical framework, we were further able to adjust for differential sampling of the population, and obtain population-level estimates of transmission flows, which is otherwise challenging. Although fishing communities have been assumed to be major sources of HIV transmission within east Africa,^10^, ^17^ our findings did not support this theory. This work highlights the use of phylogenetic approaches to not only identify but also rule out groups suspected of driving HIV spread.

Other studies have found merit in geo-targeted HIV control in African settings, particularly when hotspots comprise large numbers of people infected with HIV.^27^ For example, a study in Kenya found that geo-targeted HIV control was more effective than universal approaches even without considering the potential indirect benefits to surrounding areas.^27^ In KwaZulu-Natal, South Africa, a hotspot along a major highway was shown to be an important corridor of HIV incidence with substantial phylogenetic linkage to surrounding populations.^28^ Studies of gonorrhoea have also shown localised clustering of cases, and the impact of geo-targeted interventions to these areas.^6^ However, empirical and modelling studies of other infectious diseases identified several factors affecting the potential effects of targeting hotspots, including the connectivity between hotspots and other areas, the reproductive numbers of infections in each location, and the timing of interventions.^1^, ^3^

HIV spreads between geographical areas through two mechanisms: when infection is spread between sexual partners from different communities of residence, and when infection is spread by migration of people infected with HIV. Our results suggest the importance of both mechanisms with each accounting for approximately half of transmissions spanning fishing and inland areas. Migration was common with a quarter of the RCCS population classified as recent in-migrants (moved within 2 years). Previous studies across sub-Saharan Africa have shown that migrants who are HIV-negative are at higher risk of HIV acquisition, and migrants who are HIV-positive are less likely to be virally suppressed.^29^ Despite these findings, we did not find that migrants were at a significantly higher risk of onward transmission compared with residents. However, we found that overall, men disproportionately contributed to onward HIV transmission compared with women, and that in particular, the male to female transmission bias was greatest among men who reside (ie, did not in-migrate) in inland communities compared with women who reside in inland communities. This discrepancy in transmission by sex could partly explain why the female to male HIV prevalence ratio exceeds unity throughout sub-Saharan Africa,^30^ and emphasises the growing urgency for interventions targeted to men who are HIV-positive.

This study has limitations. First, the RCCS surveys a subset of the inland and Lake Victoria fishing communities in Rakai, Uganda. Although the sampling fraction within our communities was high, our methodological approach and hence the generalisability of our findings rests on the assumption that unobserved transmissions are missing at random within each pairwise combination of the population groups.^31^ This assumption means that including a community into the cohort did not depend on the number of HIV transmissions in or out of it, and implies that estimates of viral flow can be obtained by scaling the observed flows between RCCS communities to the populations living in inland and lakeside areas (appendix p 18). This study also did not capture short-term mobility patterns. Notably, around 30% of censused individuals did not participate in the RCCS, primarily because they were travelling for work or school, and we cannot exclude the possibility that omission of this population group might have biased our inferences. Third, we have previously reported on the quality of our deep sequence data, which was poor in some cases,^20^ although in sensitivity analyses reported in the appendix (p 21), we found that excluding lower quality reads did not impact study inferences. Fourth, people who were infected with HIV and on ART were excluded from this study potentially biasing our conclusions. However, ART coverage was higher in inland than in fishing communities over our analysis period,^13^ and so it is probable that we would have missed more transmissions from inland to fishing communities by excluding participants on ART, most of whom have suppressed viraemia. Fifth, herein we analysed around 300 phylogenetically likely source–recipient pairs, which constitute a limited sample of the actual transmission events that occurred during the observation period. Our estimates were adjusted for observed group differences in study participation, sequence sampling, and the population surveyed, but it is possible that unmeasured or unknown factors could have influenced our findings. This study is based on a population-based sample from Rakai, Uganda, between August, 2011, and January, 2015, and thus provides a snapshot of the HIV transmission dynamics between high-prevalence fishing communities and low-prevalence inland areas in this relatively recent time period. Thus, the dynamics observed in this study might not be reflective of those in the more distant past or moving forward, particularly with continued scale-up of HIV treatment and declining HIV incidence across the region, and our findings might not be applicable to hotspots in other settings.

In conclusion, we found that the HIV hotspots along Lake Victoria in Uganda, which have been hypothesised to be driving the inland epidemic in east Africa, are not a major source of HIV transmissions to the larger, lower prevalence inland populations. Lake Victoria fishing communities should be targeted for HIV control and treatment because of their high HIV burden; however, interventions in these communities are unlikely to have a broader effect on transmissions that occur inland because of their limited connectivity to inland epidemics and the relatively infrequent flow of infection between coastal and inland communities. This study cautions against equating and stigmatising high prevalence disease hotspots as population groups that drive disease spread elsewhere. More empirical studies are needed to guide modelling efforts aimed at accurately estimating the potential effect of targeted interventions.

**This online publication has been corrected. The corrected version first appeared at thelancet.com/hiv on February 6, 2020**

## Data sharing

The deep-sequence phylogenies and basic individual-level data analysed during the current study are available in the Dryad repository (DOI: 10.5061/dryad.7h46hg2). HIV-1 reads are available on reasonable request through the PANGEA consortium. Please contact project manager Lucie Abeler-Dörner (lucie.abeler-dorner@bdi.ox.ac.uk) for further details. Additional individual-level data are available on reasonable request to RHSP. Code is available on GitHub version 1.1.2 under the GNU General Public License version 3.0.
