# Correction to *Lancet HIV* 2020; published online Jan 14. https://doi.org/10.1016/S2352-3018(19)30378-9

**DOI:** 10.1016/S2352-3018(20)30041-2

**Published:** 2020-02-06

*Ratmann O, Kagaayi J, Hall M, et al. Quantifying HIV transmission flow between high-prevalence hotspots and surrounding communities: a population-based study in Rakai, Uganda.* Lancet HIV *2020; published online Jan 14. https:/doi.org/10.1016/S2352-3018(19)30378-9*—In figure 2B of this Article, the key and the legend were incorrect and have been updated. Figure 3A has also been updated and the axis labels in figure 4B have been corrected. These corrections were made as of Feb 6, 2020.

