# Supplementary material for: Quantifying HIV transmission flow between high-prevalence hotspots and surrounding communities: a population-based study in Rakai, Uganda
Source: Lancet HIV. 2020 Jan 14;7(3):e173–83. doi: 10.1016/S2352-3018(19)30378-9 (PMC7167508; doi:10.1016/S2352-3018(19)30378-9)
Supplement: Supplementary appendix [file mmc1.pdf]

THE LANCET
HIV

Supplementary appendix

This appendix formed part of the original submission and has been peer reviewed. We post it as supplied by the authors.

Supplement to: Ratmann O, Kagaayi J, Hall M, et al. Quantifying HIV transmission flow between high-prevalence hotspots and surrounding communities: a population-based study in Rakai, Uganda. *Lancet HIV* 2020; published online Jan 14. http://dx.doi.org/10.1016/S2352-3018(19)30378-9.

Supplementary Material of

Quantifying HIV transmission flow between high-prevalence hotspots and surrounding communities: a population-based study in Rakai, Uganda

*Oliver Ratmann[1], Joseph Kagaayi[2], Matthew Hall[3], Tanya Golubchick[3], Godfrey Kigozi[2], Xiaoyue Xi[1], Chris Wymant[3], Gertrude Nakigozi[2], Lucie Abeler-Dörner[3], David Bonsall[3], Astrid Gall[4], Anne Hoppe[5], Paul Kellam[6], Jeremiah Bazaale[2], Sarah Kalibbala[2], Oliver Laeyendecker[7,8], Justin Lessler[9], Fred Nalugoda[2], Larry W. Chang[2,7,9], Tulio de Oliveira[10], Deenan Pillay[5], Thomas C. Quinn[7,8], Steven J. Reynolds[2,7,8], Simon E.F. Spencer[11], Robert Ssekubugu[2], David Serwadda[2,12], Maria J. Wawer[2,9], Ronald H. Gray[2,9], Christophe Fraser[3], *M. Kate Grabowski[2,10,13], the Rakai Health Sciences Program and the Pangea HIV Consortium

[1] Department of Mathematics, Imperial College London, London SW72AZ, United Kingdom;
[2]Rakai Health Sciences Program, P.O. Box 279, Kalisizo, Old-Bukoba Road, Uganda;
[3]Oxford Big Data Institute, Li Ka Shing Centre for Health Information and Discovery, Nuffield Department of Medicine, Old Road Campus, University of Oxford, Oxford OX3 7BN, UK;
[4]European Molecular Biology Laboratory-European Bioinformatics Institute (EMBL-EBI), Wellcome Genome Campus, Hinxton, Cambridgeshire CB10 1SD, United Kingdom;
[5]Division of Infection and Immunity, University College London, London WC1E 6BT UK, United Kingdom;
[6]Department of Medicine, Imperial College London, London W12 0HS, United Kingdom;
[7]Department of Medicine, Johns Hopkins School of Medicine, Baltimore, MD 21205, USA;
[8]Division of Intramural Research, National Institute of Allergy and Infectious Diseases, NIH, Bethesda, MD 20892, USA;
[9]Department of Epidemiology, Johns Hopkins Bloomberg School of Public Health, Baltimore, MD 21205, USA;
[10]KwaZulu-Natal Research Innovation and Sequencing Platform (KRISP) College of Health Sciences, University of KwaZulu-Natal, Durban 4041, South Africa;
[11]Department of Statistics, University of Warwick, Coventry CV4 7AL, United Kingdom;
[12]Makerere University School of Public Health, P.O. Box 7072, Kampala, Uganda;
[13]Department of Pathology, Johns Hopkins School of Medicine, Baltimore, MD 21205, USA;

*Corresponding authors: oliver.ratmann@imperial.ac.uk, +44 020 759 41869; mgrabow2@jhu.edu

Supplementary Tables and Figures

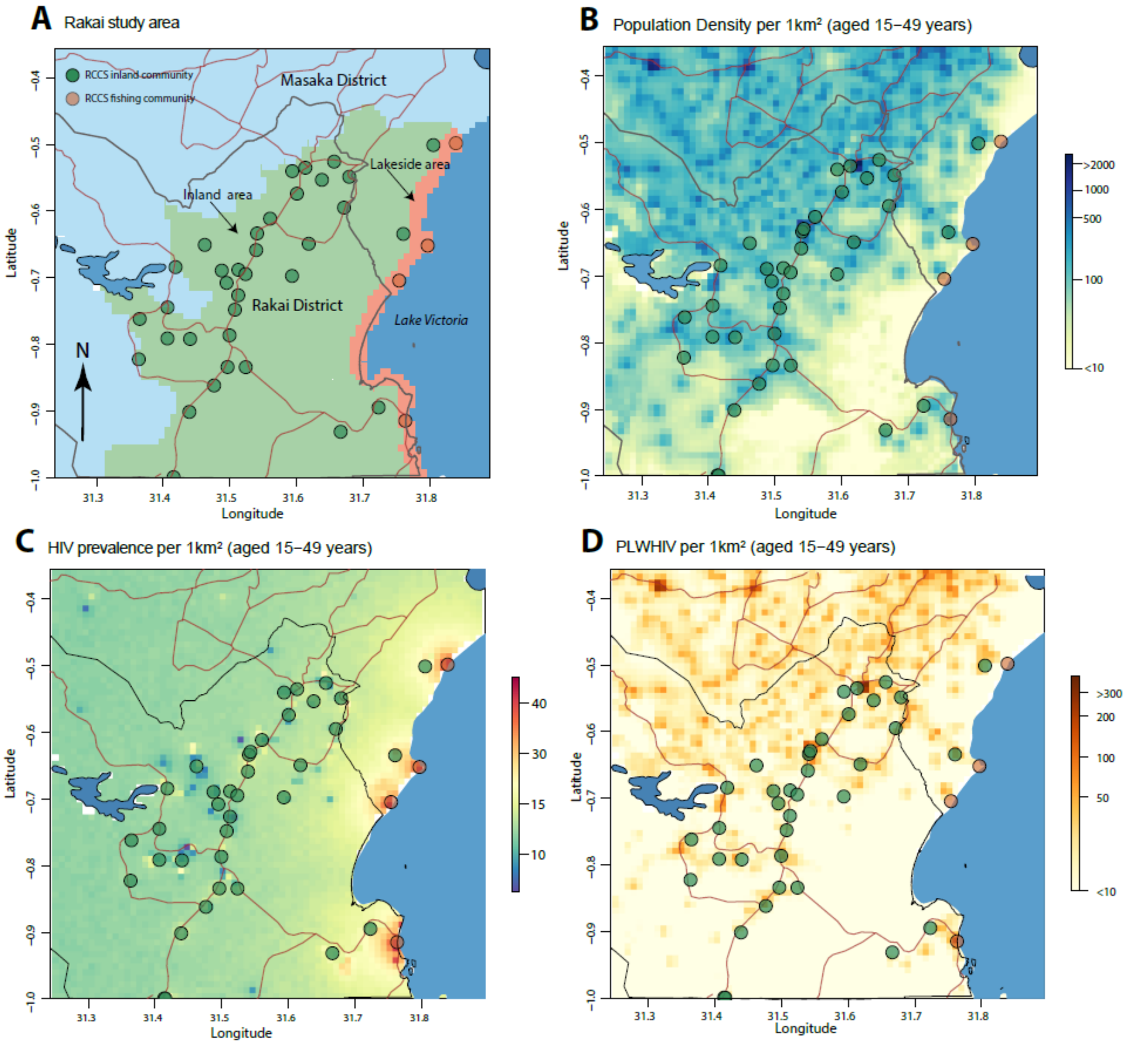

Supplementary Figure S1. Inland and fishing populations in the Rakai region. Panel A Shows RCCS inland communities in green, and Lake Victoria and fishing communities in brown. The region shown in green was designated as the RCCS inland (included administrative 2 and 3 units including RCCS communities). The RCCS included ~75·7% of populations in the lakeside area within 3km of the Lake Victoria shoreline (light brown), and ~16·2% of populations in the inland area of the Rakai region (light green). Areas classified as external in this study are shown in light blue. **Panel B** shows estimated population density of persons aged 15-49 years in the Rakai region; **Panel C** shows estimated HIV prevalence among persons aged 15-49 years in the Rakai region. **Panel D** shows the estimated number of persons living with HIV (PLHIV) aged 15-49 years in the Rakai region.

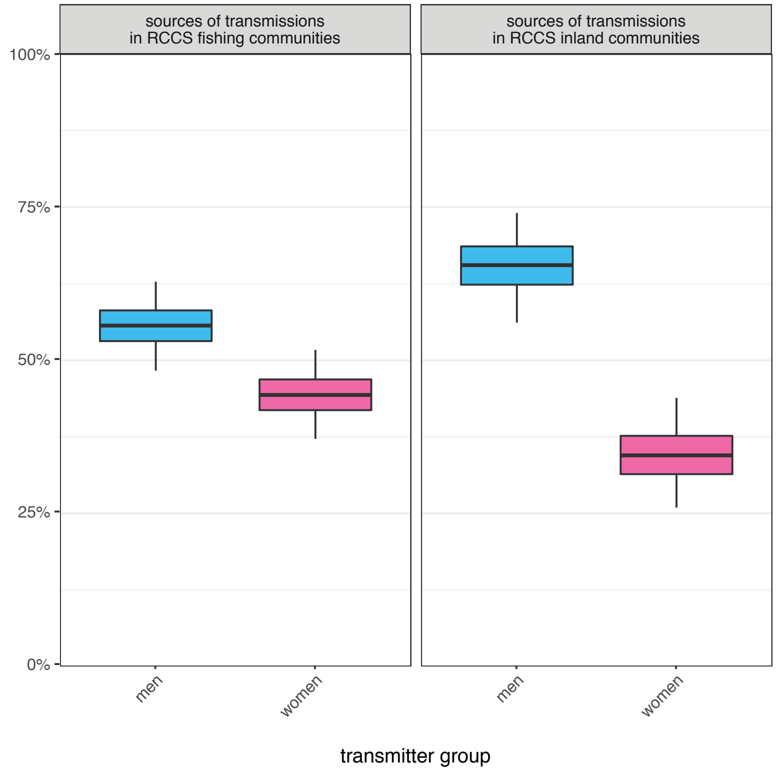

Supplementary Figure S2. Sources of transmission in by gender. To investigate the transmission bias that 60·4% (95%CrI: 54·4%-66·3%) of HIV transmissions in RCCS communities originated from men, we considered the sources of transmissions in inland and fishing communities after adjusting for sampling bias. Estimates were obtained as described in Supplementary Text S2, and adjusted for heterogeneity in participation and sequence sampling. In fishing communities, an estimated 44·3% (95%CrI: 37·2%-51·7%) of transmissions originated from women, and 55·7% (95%CrI: 48·3%-62·8%) originated from men. In inland communities, an estimated 34·5% (95%CrI: 25·9%-43·9%) of transmissions originated from women, and 65·5% (95%CrI: 56·1%-74·1%) originated from men.

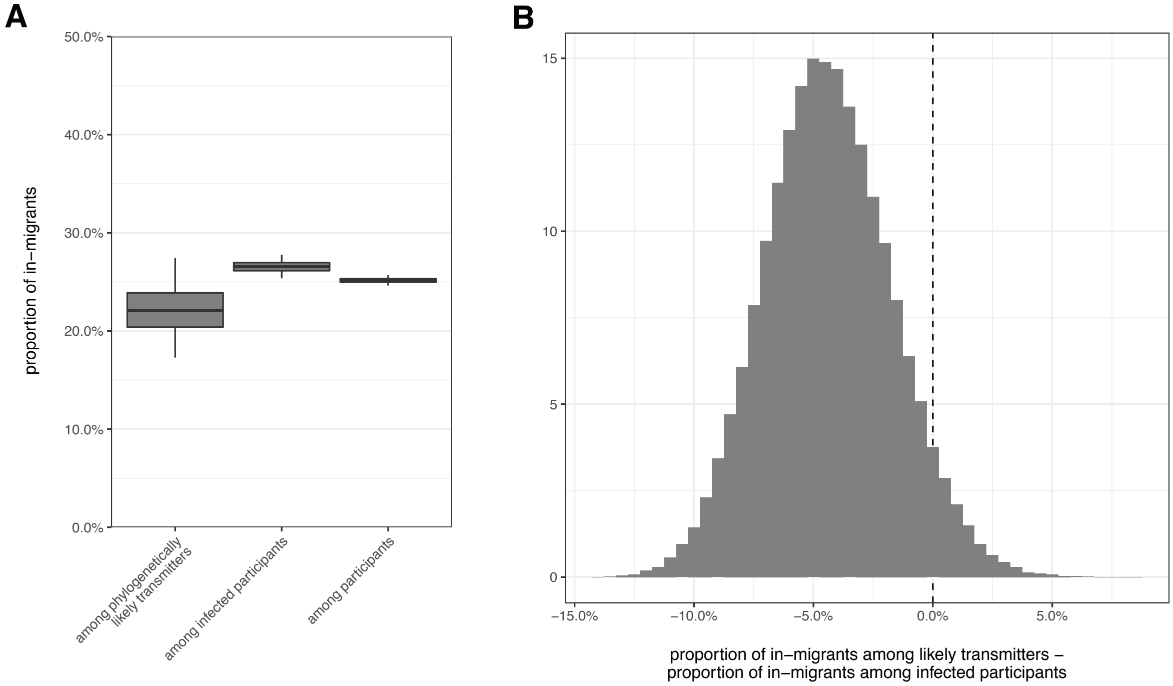

Supplementary Figure S3. Sources of transmission by migration status. To investigate the impact of in-migration on HIV transmissions in RCCS communities, we compared the proportion of in-migrants among phylogenetically likely transmitters to that among infected RCCS participants and all RCCS participants. **Panel A** shows that an estimated 22·1% (95%CrI: 17·3%-27·5%) of transmissions originated from in-migrants, compared to 26·5% (25·4%-27·8%) of infected participants who were HIV-positive in-migrants, and to 25·2% (24·6%-25·7%) of participants who were in-migrants. **Panel B** reports the posterior probability distribution of the estimated proportion of transmissions from in-migrants minus the proportion of in-migrants among infected participants. The 95% credibility interval was (-9·4%-1·1%), demonstrating that in-migrant did not contribute to the epidemic in excess of their overall representation among infected participants, and in fact likely less, although this was not statistically significant.

Supplementary Table 1. Study population and HIV-1 transmission events reconstructed with deep sequence phylogenetic analysis by migration status

**Population** (Location[*], Gender, Migration Status[†])	**Individuals eligible to participate**	**Participants**	**HIV-1 positive**	**ART-naïve[§]**	**Deep sequenced[‡]**	**Phylogenetic linkage and direction of transmission highly supported[¶]**
**Total**	**37645**	**25882**	**5142**	**3878**	**2652**	**554**
**Fishing sites, women**	**3792**	**2859**	**1352**	**1095**	**769**	**167**
Resident	2922 (77·1%)	1989 (69·6%)	957 (70·8%)	802 (73·2%)	558 (72·6%)	123 (73·7%)
Migrant from inland community	298 (7·9%)	298 (10·4%)	140 (10·4%)	110 (10%)	79 (10·3%)	14 (8·4%)
Migrant from fishing community	13 (0·3%)	13 (0·5%)	9 (0.7%)	7 (0.6%)	6 (0.8%)	2 (1.2%)
Migrant from external	465 (12·3%)	465 (16·3%)	186 (13·8%)	140 (12·8%)	98 (12·7%)	22 (13·2%)
Migrant, origin unknown	94 (2·5%)	94 (3·3%)	60 (4·4%)	36 (3·3%)	28 (3·6%)	6 (3.6%)
**Fishing sites, men**	**4737**	**3224**	**1087**	**964**	**745**	**171**
Resident	3871 (81·7%)	2358 (73·1%)	847 (77·9%)	765 (79·4%)	587 (78·8%)	140 (81·9%)
Migrant from inland community	238 (5%)	238 (7·4%)	61 (5·6%)	56 (5·8%)	40 (5·4%)	11 (6·4%)
Migrant from fishing community	19 (0·4%)	19 (0·6%)	7 (0.6%)	6 (0.6%)	6 (0.8%)	0 (0%)
Migrant from external	372 (7·9%)	372 (11·5%)	91 (8·4%)	83 (8·6%)	69 (9·3%)	14 (8·2%)
Migrant, origin unknown	237 (5%)	237 (7·4%)	81 (7·5%)	54 (5·6%)	43 (5·8%)	6 (3.5%)
**Inland communities, women**	**15154**	**10932**	**1797**	**1156**	**678**	**112**
Resident	12146 (80·2%)	7924 (72·5%)	1248 (69·4%)	755 (65·3%)	473 (69·8%)	87 (77·7%)
Migrant from inland community	1372 (9·1%)	1372 (12·6%)	258 (14·4%)	195 (16·9%)	106 (15·6%)	16 (14·3%)
Migrant from fishing community	18 (0·1%)	18 (0·2%)	5 (0.3%)	5 (0.4%)	2 (0.3%)	2 (1.8%)
Migrant from external	1238 (8·2%)	1238 (11·3%)	185 (10·3%)	145 (12·5%)	72 (10·6%)	6 (5.4%)
Migrant, origin unknown	380 (2·5%)	380 (3·5%)	101 (5·6%)	56 (4·8%)	25 (3·7%)	1 (0.9%)
**Inland communities, men**	**13962**	**8867**	**906**	**663**	**460**	**104**
Resident	12195 (87·3%)	7100 (80·1%)	725 (80%)	518 (78·1%)	374 (81·3%)	87 (83·7%)
Migrant from inland community	690 (4·9%)	690 (7·8%)	81 (8·9%)	65 (9·8%)	36 (7·8%)	9 (8.7%)
Migrant from fishing community	9 (0.1%)	9 (0.1%)	3 (0.3%)	3 (0.5%)	3 (0.7%)	2 (1.9%)
Migrant from external	635 (4·5%)	635 (7·2%)	45 (5%)	41 (6·2%)	18 (3·9%)	5 (4.8%)
Migrant, origin unknown	433 (3·1%)	433 (4·9%)	52 (5·7%)	36 (5·4%)	29 (6·3%)	1 (1%)

* RCCS communities on the shore of Lake Victoria were classified as fishing site, and all others as inland communities.
† Individuals who in-migrated into an RCCS community in the two years before their first survey visit were classified as an in-migrant, and otherwise as resident. Origins of migration were geo-located from interview data.
§ Infected individuals who did not self-report use of ART.
‡ Infected ART-naïve individuals who had deep sequences at sufficient quality for analysis, defined as reads of length at least 250nt that covered a minimum of 750nt of the HIV-1 genome at a sequencing depth of 30X.
¶ Sequenced individuals who were phylogenetically close, adjacent, and ancestral in the same direction to another individual in viral deep-sequence phylogenies across 60% of the HIV-1 genome.

Supplementary Table 2. HIV-1 transmissions among RCCS communities by source location

Source population	Recipient population	Estimated contribution to overall HIV-1 transmissions among RCCS communities * (mean, 95% credibility interval of posterior density)	Predicted contribution to overall HIV-1 transmission among Rakai subdistricts ** (mean, 95% credibility interval of posterior predictive density)
**Overall**			
Fishing sites	Fishing sites	76·4% (69·7%-82·4%)	54·8% (42·2%-69%)
Inland communities	Fishing sites	13·4% (8·8%-19·1%)	45·2% (31%-57·8%)
External to RCCS	Fishing sites	10% (6·1%-15·2%)	--
Fishing sites	Inland communities	8.3% (4%-14·9%)	1.9% (0.7%-3.9%)
Inland communities	Inland communities	85·4% (77·6%-91·4%)	98·1% (96·1%-99·3%)
External to RCCS	Inland communities	5.9% (2.3%-11·7%)	--
**By gender**			
M, Fishing sites	F, Fishing sites	78·5% (69·4%-86·1%)	54% (37·2%-73·7%)
M, Inland communities	F, Fishing sites	12·9% (7·1%-20·9%)	46% (26·3%-62·8%)
M, External to RCCS	F, Fishing sites	8.1% (3.8%-14·8%)	--
M, Fishing sites	F, Inland communities	11·6% (5·3%-21%)	2.6% (0.9%-5.7%)
M, Inland communities	F, Inland communities	82·2% (71·7%-90·2%)	97·4% (94·3%-99·1%)
M, External to RCCS	F, Inland communities	5.7% (1.7%-13·1%)	--
F, Fishing sites	M, Fishing sites	74% (63·5%-83·1%)	56·8% (38·9%-77·2%)
F, Inland communities	M, Fishing sites	13·6% (7·2%-22·4%)	43·2% (22·8%-61·1%)
F, External to RCCS	M, Fishing sites	11·9% (5·9%-20·6%)	--
F, Fishing sites	M, Inland communities	1.6% (0.1%-8.1%)	0.4% (0%-2.7%)
F, Inland communities	M, Inland communities	92·3% (80·4%-98·1%)	99·6% (97·3%-100%)
F, External to RCCS	M, Inland communities	5.4% (0.8%-16·6%)	--

* Estimates based on phylogenetically reconstructed events, and adjusted for participation and sequencing differences via Bayesian multi-level model; see Supplementary Text S2. ** Predictions based on fitted Bayesian multi-level model, and extrapolating from eligible individuals who live in RCCS communities to the inland and fishing areas shown in Figure 1A; see Supplementary Text S3.

Supplementary Table 3. HIV-1 transmissions among RCCS communities by recipient location

Source population	Recipient population	Estimated contribution to overall HIV-1 transmissions among RCCS communities * (mean, 95% credibility interval of posterior density)	Predicted contribution to overall HIV-1 transmission among Rakai subdistricts ** (mean, 95% credibility interval of posterior predictive density)
**Overall**			
Fishing sites	Fishing sites	92·8% (87·1%-96·5%)	75·2% (59·2%-89·1%)
Fishing sites	Inland communities	7.2% (3.5%-12·9%)	24·8% (10·9%-40·8%)
Inland communities	Fishing sites	18% (11·9%-25·5%)	4.6% (2.5%-7.6%)
Inland communities	Inland communities	82% (74·5%-88·1%)	95·4% (92·4%-97·5%)
External to RCCS	Fishing sites	70·3% (49·3%-87%)	--
External to RCCS	Inland communities	29·7% (13%-50·7%)	--
**By gender**			
M, Fishing sites	F, Fishing sites	89% (80·1%-94·9%)	66·2% (46·8%-85·4%)
M, Fishing sites	F, Inland communities	11% (5·1%-19·9%)	33·8% (14·6%-53·2%)
M, Inland communities	F, Fishing sites	15·8% (8·6%-25·5%)	4.3% (1.8%-8.4%)
M, Inland communities	F, Inland communities	84·2% (74·5%-91·4%)	95·7% (91·6%-98·2%)
M, External to RCCS	F, Fishing sites	63% (34·5%-87%)	--
M, External to RCCS	F, Inland communities	37% (13%-65·5%)	--
F, Fishing sites	M, Fishing sites	98·8% (94%-99·9%)	94·8% (71·2%-100%)
F, Fishing sites	M, Inland communities	1.2% (0.1%-6%)	5.2% (0%-28·8%)
F, Inland communities	M, Fishing sites	21·1% (11·2%-34%)	4.9% (1.9%-10·1%)
F, Inland communities	M, Inland communities	78·9% (66%-88·8%)	95·1% (89·9%-98·1%)
F, External to RCCS	M, Fishing sites	80% (49·3%-96·7%)	--
F, External to RCCS	M, Inland communities	20% (3·3%-50·7%)	--

* Estimates based on phylogenetically reconstructed events, and adjusted for participation and sequencing differences via Bayesian multi-level model; see Supplementary Text S2. ** Predictions based on fitted Bayesian multi-level model, and extrapolating from eligible individuals who live in RCCS communities to the inland and fishing areas shown in Figure 1A; see Supplementary Text S3.

Supplementary Table 4. Rakai sub-districts with RCCS surveillance over the study period.

Geographic area	Gender	Estimated population, ages 15-49 years	Estimated infected population, ages 15-49 years	Census-eligible population in RCCS communities, ages 15-49 years	Study participants in RCCS communities, ages 15-49 years	Infected population in RCCS communities, ages 15-49 years
		#	# (%HIV+)	#	#	# (%HIV+)
lakeside	F	2981	837 (28·1%)	3792	2859	1352 (47·3%)
lakeside	M	2655	558 (21·0%)	4737	3224	1087 (33·7%)
inland	F	98476	14927 (15·1%)	15154	10932	1797 (16·4%)
inland	M	81506	9951 (12·2%)	13962	8867	906 (10·2%)

Supplementary Text S1 Rakai Community Cohort Study

S1.1 RCCS Recruitment and follow-up

The Rakai Community Cohort Study (RCCS), conducted by the Rakai Health Sciences Program (RHSP), is an open, population-based, multi-community cohort of individuals aged 15-49 years. To identify eligible cohort participants, a household census enumerates all persons by gender, age, and duration of residence, irrespective of age, and whether they are present or currently absent. Eligible individuals are then invited to come to a central hub in the community for RCCS consent and enrolment. At the hub, individuals undergo group consent procedures (information is provided to a group at a time), followed by individual consent which is conducted in private by a trained RCCS interviewer/counsellor. Two attempts are made to contact individuals at their home if they were censused and eligible but who do not present at the hubs for survey. Mobile phone outreach is also performed for survey participants from prior rounds who are not present at subsequent surveys. There are no specific incentives for follow-up given, but all participants are compensated for time and travel.

For this study, participants were enrolled between August 10, 2011 and January 30, 2015. Figure S4 illustrates the distribution of first survey visit times of participants in inland and fishing communities, showing that the two populations were surveyed concurrently.

S1.2 RCCS survey procedures

Each RCCS survey round collects detailed interview data (sociodemographic, behavioral, sexual network, health care utilization, pregnancy and childbearing, health status) consenting residents aged 15-49. Interviews are conducted in private by trained same sex interviews in the local language, Luganda, with direct data entry into password protected encrypted mobile PCs.

As part of the survey, all RCCS participants are offered free voluntary counseling and HIV testing if they previously tested HIV-negative in a prior RCCS survey or their HIV status is unknown. The vast majority (>90%) of participants over the lifetime of the cohort consent to be tested, and to receive their results. A small percentage (<5%) agree to be tested but choose not to receive their HIV results. HIV rapid testing is performed using a validated algorithm, and results are returned to participants immediately through on-site post-test counselors.

All consenting participants, irrespective of HIV status, are also provide a venous blood sample for storage/future testing, including viral phylogenetic studies. Blood is collected in EDTA tubes, and after collection, which occurs at the hub, specimens are stored in a cool box until transport to the central RHSP laboratory. After specimen arrival to the central lab, specimens are centrifuged, and plasma is separated into 1 ml aliquots for storage. Aliquots are labelled with participants' unique alphanumeric ID, and stored at -80ºC in a designated freezer facility on site. In case of power failure in the grid, the freezer facility is connected to a generator house with two backup 200 KVA and one 150 KVA generators, UPSs, 24 inverters, and a 20-battery backup for uninterrupted power.

S1.3 Viral sequencing

HIV-1 deep sequences were generated from blood samples of HIV infected study participants who did not report ART use. This selection criterion was motivated by the fact that self-reported ART use reflected actual ART use with high specificity and sensitivity in a previous validation study(1), and that 90% of individuals who reported ART use had suppressed virus below 1,000 copies per mL of plasma(2), below which viral deep sequencing was not possible with our protocol(3). If an individual participated in more than one survey over the observation period and they reported no ART use at multiple visits, only the sample at the initial visit at which they reported no ART use was scheduled for sequencing. If an individual was observed multiple times and initially reported ART use but at a later visit did not, the sample of the first visit at which they did not report ART use during the observation period was scheduled for sequencing. Thus, one sample per participant was scheduled for sequencing, and it was the first visit at which they did not report ART use during the observation period. Samples scheduled for sequencing were shipped to University College London Hospital, London, United Kingdom for viral RNA extraction. RNA extraction was automated on QIAsymphony SP workstations with the QIA- symphony DSP Virus/Pathogen Kit (Cat. No. 937036, 937055; Qiagen, Hilden, Germany), followed by one-step reverse transcription polymerase chain reaction (RT-PCR). Deep-sequencing was performed on Illumina MiSeq and HiSeq instruments in the DNA pipelines core facility at the Wellcome Trust Sanger Institute, Hinxton, United Kingdom.

2,652 individuals had viral deep-sequences generated that satisfied minimum quality criteria for phylogenetic analysis(4). Figure S5 illustrates the sequence sampling times of these individuals, indicating that the sampling of the populations in fishing and inland communities overlapped in time.

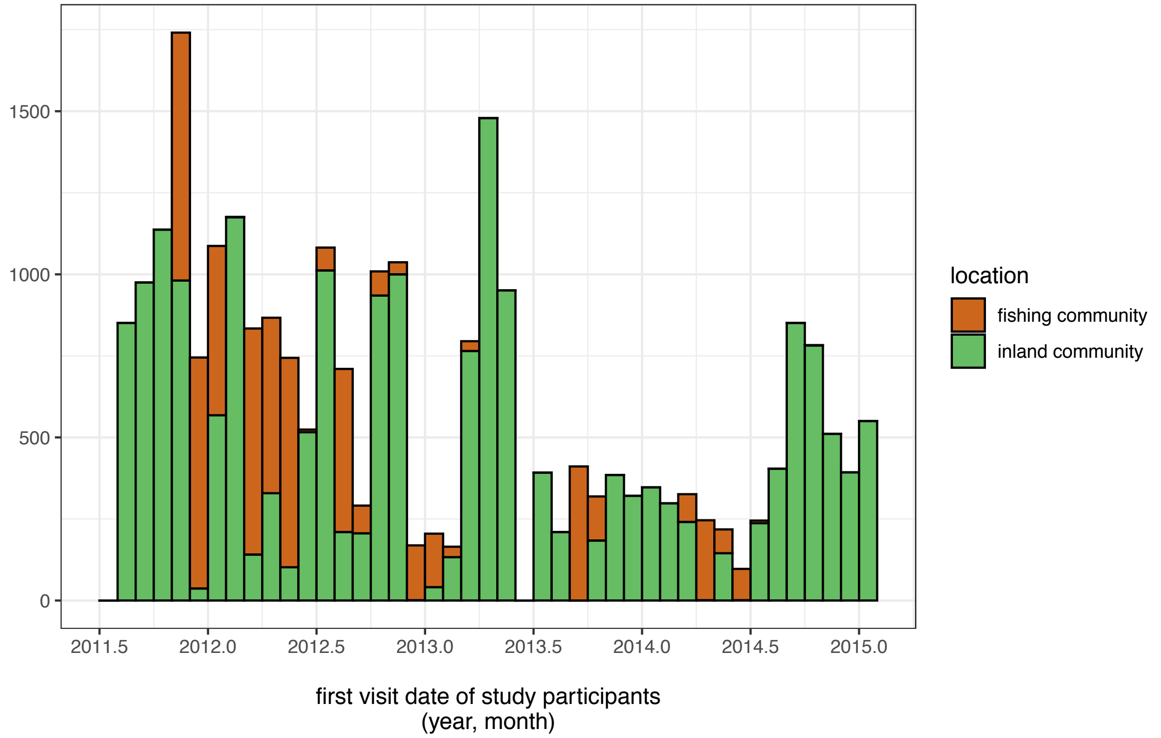

Supplementary Figure S4. Survey dates in inland and fishing communities. 25,882 individuals in 40 communities participated in the Rakai Community Cohort Study from August 10, 2011 to January 30, 2015. The histogram shows the number of study participants in inland communities (green), and fishing communities (brown) by month in the observation period. In inland communities, the first and last visit dates were respectively August 10, 2011 and January 30, 2015. In fishing communities, the first and last visit dates were respectively November 4, 2011 and October 2, 2014.

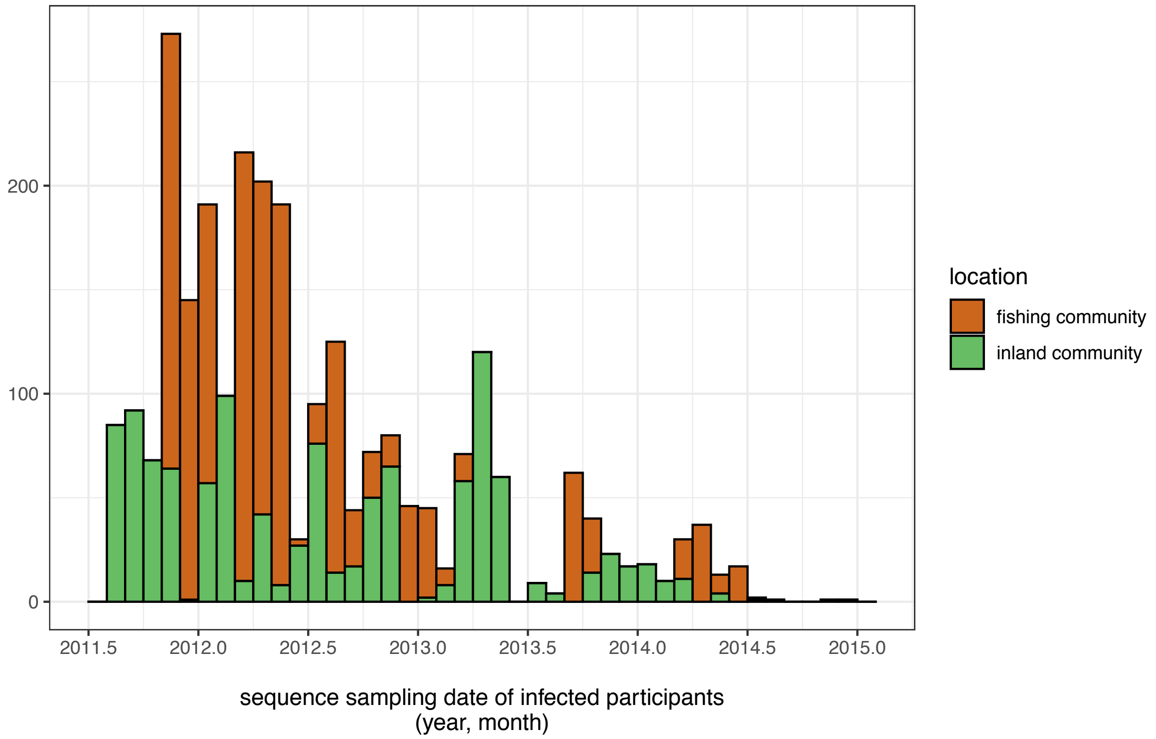

Supplementary Figure S5. Sampling dates associated with viral sequences in inland and fishing communities. From 2,652 individuals, viral sequences could be obtained that satisfied minimum criteria on read length and read depth(4). Overall, the sequences were obtained from participants visited from August 10, 2011 to December 3, 2014. In inland communities, the respective dates were August 10, 2011 to December 3, 2014. In fishing communities, the respective dates were November 21, 2011 to July 3, 2014.

Supplementary Text S2 Statistical analysis of transmission flows between surveyed communities

S2.1 Source attribution model

Input data from phyloscanner deep sequence analysis

2,652 individuals of 3,878 HIV-infected individuals who did not report ART use were deep-sequenced satifying minimum quality criteria for phylogenetic analysis, and the phyloscanner software was used to reconstruct their viral phylogenetic relationships(5). Full details are described in Ratmann et al.(4). Briefly: in a first stage, pairs of individuals who had phylogenetically close virus across the HIV-1 genome were identified. Individuals were randomly assigned to batches of individuals, and phyloscanner was run on viral deep-sequence data from individuals in each pairwise combination of batches. This allowed identification of all pairs of individuals with phylogenetically close virus. Potential transmission networks were then constructed by grouping individuals who had phylogenetically close virus to at least one other individual. In a second stage, phyloscanner was run on viral deep-sequence data from all individuals in a potential transmission network, and sequences from the ~10 most phylogenetically closely related individuals not in the potential network that acted as controls. The topology of deep-sequence trees and phylogenetic distance were used to confirm membership of individuals in a transmission network, and to estimate the direction of transmission within networks. Each network was described with two adjacency matrices $\boldsymbol{L}$ and $\boldsymbol{D}$ that quantified respectively the strength of phylogenetic evidence for direct transmission (linkage) between two individuals in the network, and the strength of phylogenetic evidence for the direction of transmission between two individuals in the network. The cell entry L_{ij} was obtained by counting the number of deep-sequence phylogenies with evidence for linkage across the HIV-1 genome, and then adjusting the raw count for the extent of overlap in the read alignments from which the deep-sequence phylogenies were reconstructed. The cell entry D_{ij} was obtained by counting the number of deep-sequence phylogenies with evidence for transmission direction from i to j, and then by adjusting the raw count for the extent of overlap in the read alignments from which the deep-sequence phylogenies were reconstructed. Two individuals were defined as a phylogenetically likely transmission pair with strong support for the direction of transmission (source-recipient pair) if $L_{ij}/K > c$ and $D_{ij}/L_{ij} > c$, where $c = 0.6$. 293 source-recipient pairs were reconstructed.

Definition of inland, fishing, and external populations for source attribution

Figure 1A shows the 36 inland and four fishing communities that were part of the RCCS between August 2011 and January 2015. All study participants and source-recipient pairs resided in one of these communities at time of survey, of whom a quarter had migrated into RCCS communities within two years before to study visit (see main text). To account for these population movements, inland and fishing populations were defined more broadly. The northernmost and southernmost RCCS communities were located at latitudes -0·406 and -0·999 respectively. Fishing populations were defined to be located within 3km to the shores of Lake Victoria within latitudes -0·406 and -0·999. The 3km range was chosen so that the lakeside area contained all households belonging to Lake Victoria fishing communities, and so that the geographic center of all inland communities was not in the lakeside area. Inland populations were defined to be sub-districts where RCCS surveillance took place within the same latitude range, with the exception of fishing populations. External populations were defined to be outside sub-districts where RCCS surveillance took place, or beyond latitudes -0·406 and -0·999. Supplementary Figure S1 illustrates the locations of inland, fishing and external populations.

Crude estimate of transmission flows

The aim of analysis is to estimate the population-level proportion of transmissions π_{ab} from population sub-group a to population sub-group b. Every individual is assumed to be part of one stratum. In this study, we focused on estimating transmission flows by location, with the population stratified either in three groups (fishing, inland or external populations), or stratified in six groups (fishing:men, fishing:women, inland:men, inland:women, external:men, external:women). To introduce notation, suppose there are in total z_{ab} transmissions from group a to group b, of which n_{ab} are observed in a cross-sectional population-based sample, with corresponding totals $Z = \sum_{a,b} z_{ab}$, and $N = \sum_{a,b} n_{ab}$. Table 1 left column reports the number of observed transmission events n_{ab}, and the crude estimate $\tilde{\pi}_{ab} = n_{ab}/N$. Under the assumption that individuals are sampled at random with probability ξ_a in stratum a, then on expectation

$$E[\tilde{\pi}_{ab}] = (z_{ab}\xi_a\xi_b)/(\sum_{c,d} z_{cd}\xi_c\xi_d)$$

and $\tilde{\pi}_{ab}$ is an unbiased estimator of π_{ab} if the sampling probabilities are homogeneous, i.e. ξ_a is independent of a for all strata a and just a constant. This is usually not the case.

Bayesian data augmentation model

For the case that sampling probabilities are not homogeneous, we developed the following Bayesian multi-level model to obtain sampling-adjusted estimates of π_{ab}. The central assumption we make is that prior information on ξ_a are available, for example through enumeration and surveillance of the entire study population. Define the vector $\boldsymbol{n} = (n_{ab})$ to be the number of observed transmission flow counts for all pairwise strata combinations considered, $n_{ab} \geq 0$, e.g. fishing->fishing, fishing->inland, inland->fishing, inland->inland, or men->women, and women->men. Due to population movement, the basic scenario that we consider includes fishing->fishing, fishing->inland, inland->fishing, inland->inland, external->fishing, and external->inland, and so the length of $\boldsymbol{n}$ is $L = 6$. Also denote the vectors $\boldsymbol{z} = (z_{ab})$, $\boldsymbol{\pi} = (\pi_{ab})$, $\boldsymbol{\xi} = (\xi_a)$. Assuming that source-recipient pairs are independent, we consider the model

$$\boldsymbol{z} \sim Multinomial(Z, \boldsymbol{\pi})$$
$$n_{ab} \sim Binomial(z_{ab}, \xi_a \xi_b)$$
$$\xi_a \sim p(\xi_a)$$
$$Z \sim p(Z)$$
$$\boldsymbol{\pi} \sim p(\boldsymbol{\pi})$$

where the counts n_{ab} are observed, $\boldsymbol{\pi}$ are the target parameters, and $\boldsymbol{z}$, Z, ξ_a are latent parameters. The Bayesian model allows incorporation of information on the sampling probability for all strata through the prior distributions $p(\xi_a)$, for example through Beta distributions that are centered at particular values and with particular precision. The prior distribution on the total number of transmissions, $p(Z)$, can also be specified based on available sampling information, by setting $p(Z)$ to a truncated Poisson distribution with lower limit N and median around N divided by the average sampling fraction of the population. The prior distribution on $\boldsymbol{\pi}$ was set to an uninformative conjugate Dirichlet distribution with constant hyper-parameters $\boldsymbol{\lambda} = (\lambda_{ab})$, $\lambda_{ab} = 0.8/L$. The joint posterior distribution $p(\boldsymbol{\pi}, \boldsymbol{z}, Z, \boldsymbol{\xi} \mid \mathbf{n})$ can be estimated via Markov Chain Monte Carlo, including high-dimensional cases when the number of transmission flows to be estimated exceeds 100. The algorithm is available at https://github.com/BDI-pathogens/phyloscanner/phyloflows, version 1.1.0. The main simplifying assumption of this model is that phylogenetically reconstructed transmission events are assumed to be independent in the Multinomial data likelihood, which can be inappropriate when reconstructed transmission networks are large. When the large majority of reconstructed transmission networks consists of two individuals as in the Rakai data, the independence assumption is not overly restrictive.

S2.2 Application to Rakai data

Overall model specification

We applied the source attribution model in section S2.1 to estimate HIV transmission flows between inland and fishing communities of the RCCS. The phylogenetic data consisted of 293 source-recipient pairs that were reconstructed through deep-sequence viral phylogenetic analysis of a population-based sample of 2,652 RCCS participants. Following extensive previous characterization of the study population (2, 6, 7), the model in section S2.1 was specified in terms of strata a that were a combination of gender (male, female), age bracket (15-24, 25-34, 35-50 years), migration status (in-migration in 2 years before first RCCS visit in study period, resident otherwise), and community type (agrarian, trading, fishing). This detailed description was chosen to accommodate variation in participation and sequence sampling rates in the cohort.

Modelling variation in participation rates

Figure S6 illustrates participation rates (#participants/#eligible) by migration status, gender, and age bracket. Results for each community are represented as a point. There were significant overall differences in participation rates by gender and age, with variation across RCCS communities. The differences were described in a Bayesian Beta-Binomial logistic regression model including all interaction terms between gender, age bracket, and migration status,

$$k_i^{par} \sim BetaBinomial(\xi_i^{par}, n_i^{eli}, \phi)$$
$$logit(\xi_i^{par}) = \beta_0 + \beta_1 F_i + \beta_2 G_i A_{1i} M_i + \beta_3 G_i A_{2i} M_i + \beta_4 G_i M_i +$$
$$\beta_5 (1 - G_i) A_{1i} M_i + \beta_6 (1 - G_i) A_{2i} M_i + \beta_7 (1 - G_i) M_i +$$
$$\beta_8 G_i A_{1i} (1 - M_i) + \beta_9 G_i A_{2i} (1 - M_i) + \beta_{10} G_i (1 - M_i) +$$
$$\beta_{11} (1 - G_i) A_{1i} (1 - M_i) + \beta_{12} (1 - G_i) A_{2i} (1 - M_i)$$
$$\beta_0 \sim Normal(0,100)$$

$$\beta_j \sim Normal(0,10) \text{ for } j = 1, \dots, 12$$

with data

k_i^{par} RCCS participants in stratum i
n_i^{eli} census eligible individuals in stratum i
G_i gender status in stratum i (male=1, female=0)
F_i fishing community status in stratum i (yes=1, no=0)
T_i trading community status in stratum i (yes=1, no=0)
M_i inmigration status in stratum i (yes=1, no=0)
A_{1i} age bracket 15-24 years in stratum i (yes=1, no=0)
$A_{2\square}$ age bracket 25-34 years in stratum i (yes=1, no=0)

and estimated parameters

ξ_i^{par} participation probability in stratum i
ϕ overdispersion parameter
β_j fixed effects regression parameters, $j = 0, \dots, 12$.

Similar versions of the model with different interaction terms were fitted with Stan version 2.19 (8), and the final version reported above was chosen based on best WAIC. The fixed effects parameters $\beta_0, \beta_5, \beta_8, \beta_9, \beta_{10}, \beta_{11}, \beta_{12}$ had marginal posterior distributions with 95% credibility intervals excluding zero. Monte Carlo samples of the marginal posterior distributions of ξ_i^{par} were used for sampling adjusted source attribution as described below.

Modelling variation in sequence sampling rates

Figure S7 illustrates sequence sampling rates (#sequenced at minimum quality criteria/#infected and not reporting ART use) by migration status, gender, and age bracket. Results for each community are represented as a point. There were significant overall differences in participation rates by gender and age, with variation across RCCS communities. The differences were described in a Bayesian Binomial logistic regression model of the form

$$k_i^{seq} \sim Binomial\left(\xi_i^{seq}, n_i^{noARTuse}\right)$$
$$logit\left(\xi_i^{par}\right) = \beta_0 + \beta_1 G_i + \beta_2 F_i + \beta_3 T_i + \beta_4 M_i + \beta_5 A_{1i} + \beta_6 A_{2i}$$
$$\beta_0 \sim Normal(0,100)$$
$$\beta_j \sim Normal(0,10) \text{ for } j = 1, \dots, 6$$

with data and estimated parameters

k_i^{seq} individuals of whom virus was deep-sequenced at minimum quality criteria in stratum i
$n_i^{noARTuse}$ infected individuals who did not report ART use in stratum i
ξ_i^{seq} sequencing probability in stratum i

and all other variables defined as for the participation rate analysis. Similar versions of the model were fitted with Stan version 2.19 (8). The final version reported above was chosen based on best WAIC. Models with overdispersion and/or interaction terms had worse WAIC values. The fixed effects parameters $\beta_0, \beta_1, \beta_2, \beta_3, \beta_4, \beta_4$, β_5 had marginal posterior distributions with 95% credibility intervals excluding zero. Monte Carlo samples of the marginal posterior distributions of ξ_i^{seq} were used for sampling adjusted source attribution as described below.

Specification of the sampling variable

For stratum a, overall sampling was modelled as the product of study participation and, subsequently, sequencing if participants were infected, $p(\xi_a) = p\left(\xi_a^{par}\right) p\left(\xi_a^{seq}\right)$, where $p\left(\xi_a^{par}\right)$ is the marginal posterior distribution of participation rates under the above Beta-Binomial-logistic model, and $p\left(\xi_a^{seq}\right)$ is the marginal posterior distribution of sequencing rates under the above Binomial-logistic model. To fit the Bayesian data augmentation model of section S2.1, Monte Carlo samples from $p(\xi_a)$ are required. These were obtained by drawing Monte Carlo samples from $p\left(\xi_a^{par}\right)$ and $p\left(\xi_a^{seq}\right)$ that were obtained with Stan, and multiplying both samples.

Computational inference

To adjust for sampling differences by gender, age, migration status, and community type, we extended the flow vector $\boldsymbol{\pi}$ of size $L = 6$ to account for a finer stratification of the population by sampling groups. Based on our stratification by gender, age bracket, migration status, and community type, the resulting flow vector $\boldsymbol{\pi}$ had length $L = 576$, and captured, for example, the proportion of transmissions from resident men aged 25-29 in inland communities to resident women aged 15-24 in inland communities. Most entries in the observation vector $\boldsymbol{n} = (n_{ab})$ were zero. However because of incomplete sampling, the corresponding (unobserved) actual transmission counts z_{ab} were often non-zero, and under the model of section S2.1 the probabilities that the actual (unobserved) transmission counts were non-zero differed for each entry of $\boldsymbol{z} = (z_{ab})$ because the sampling probabilities ξ_a and ξ_b differed in each case. The joint posterior distribution p($\boldsymbol{\pi}, \boldsymbol{z}$, Z, $\boldsymbol{\xi}$ | **n**) was numerically estimated with the MCMC algorithm in Section S2.1 in 4.8 million MCMC iterations. Figure S8 reports traceplots of the primary parameter of interest, $\boldsymbol{\pi}$. Convergence was assessed with the Gelman-Rubin statistic, and mixing was assessed in terms of effectice sample size, as calculated with the coda R package version 0.19-2. Numerical convergence was achieved in a burn-in period of 240e3 iterations, and the effective sample sizes from the marginal posterior densities were all above 10,000, confirming that inference of $\boldsymbol{\pi}$ on the Rakai data set was computationally feasible.

Reported quantities

To characterize transmission dynamics between inland and fishing RCCS communities, the following quantities were derived as summary measures from MCMC output:

quantity	*symbol*	*definition*
transmission flows between inland and fishing communities in %	$\pi_{FF}, \pi_{IF}, \pi_{[illegible]}\ \pi_{II}, \pi_{EI}, \pi_{EF}$ where F denotes fishing communities, I inland communities, E external introductions through inmigration, with π_{FF}+π_{IF}+π_{FI}+$\pi_{II} + \pi_{EI} + \pi_{EF} = 1$	$\pi_{FF} = \sum_{a \in F, b \in F} \pi_{ab}$, where π_{ab} is defined above as the proportion of transmissions from stratum a to stratum b; i.e. MCMC output was aggregated across strata in fishing communities. Calculations for $\pi_{IF}, \pi_{FI}, \pi_{II}$, π_{EI}, π_{EF} were done analogously.
transmission flow ratio	γ	$\gamma = \pi_{IF}/\pi_{FI}$
sources of infection in fishing communities in %	$\delta_F^F, \delta_I^F, \delta_E^F$	$\delta_F^F = \pi_{FF}/(\pi_{FF} + \pi_{IF} + \pi_{EF})$, $\delta_I^F = \pi_{IF}/(\pi_{FF} + \pi_{IF} + \pi_{EF})$, $\delta_E^F = \pi_{EF}/(\pi_{FF} + \pi_{IF} + \pi_{EF})$
sources of infection in inland communities in %	$\delta_I^I, \delta_F^I, \delta_E^I$	$\delta_I^I = \pi_{II}/(\pi_{II} + \pi_{FI} + \pi_{EI})$, $\delta_F^I = \pi_{FI}/(\pi_{II} + \pi_{FI} + \pi_{EI})$, $\delta_E^I = \pi_{EI}/(\pi_{II} + \pi_{FI} + \pi_{EI})$
recipients of infection from fishing communities in %	ω_F^F, ω_I^F	$\omega_F^F = \pi_{FF}/(\pi_{FF} + \pi_{FI})$, $\omega_I^F = \pi_{FI}/(\pi_{FF} + \pi_{FI})$
recipients of infection from inland communities in %	ω_F^I, ω_I^I	$\omega_F^I = \pi_{FF}/(\pi_{IF} + \pi_{II})$, $\omega_I^I = \pi_{IF}/(\pi_{IF} + \pi_{II})$
recipients of infection from inmigration in %	ω_F^E, ω_I^E	$\omega_F^E = \pi_{EF}/(\pi_{EF} + \pi_{EI})$, $\omega_I^E = \pi_{EI}/(\pi_{EF} + \pi_{[illegible]}$

Reported error bars are 95% highest posterior density intervals of the marginal posterior densities of the above variables. Estimates stratified by inland and fishing RCCS communities and gender were calculated analogously. Table 1 reports estimated transmission flows between fishing and inland communities. Figure 3 reports the estimated transmission flow ratio. Table S2 reports estimated sources of infection in fishing communities and in inland communities. Table S3 reports estimated recipients of infection in fishing communities and in inland communities.

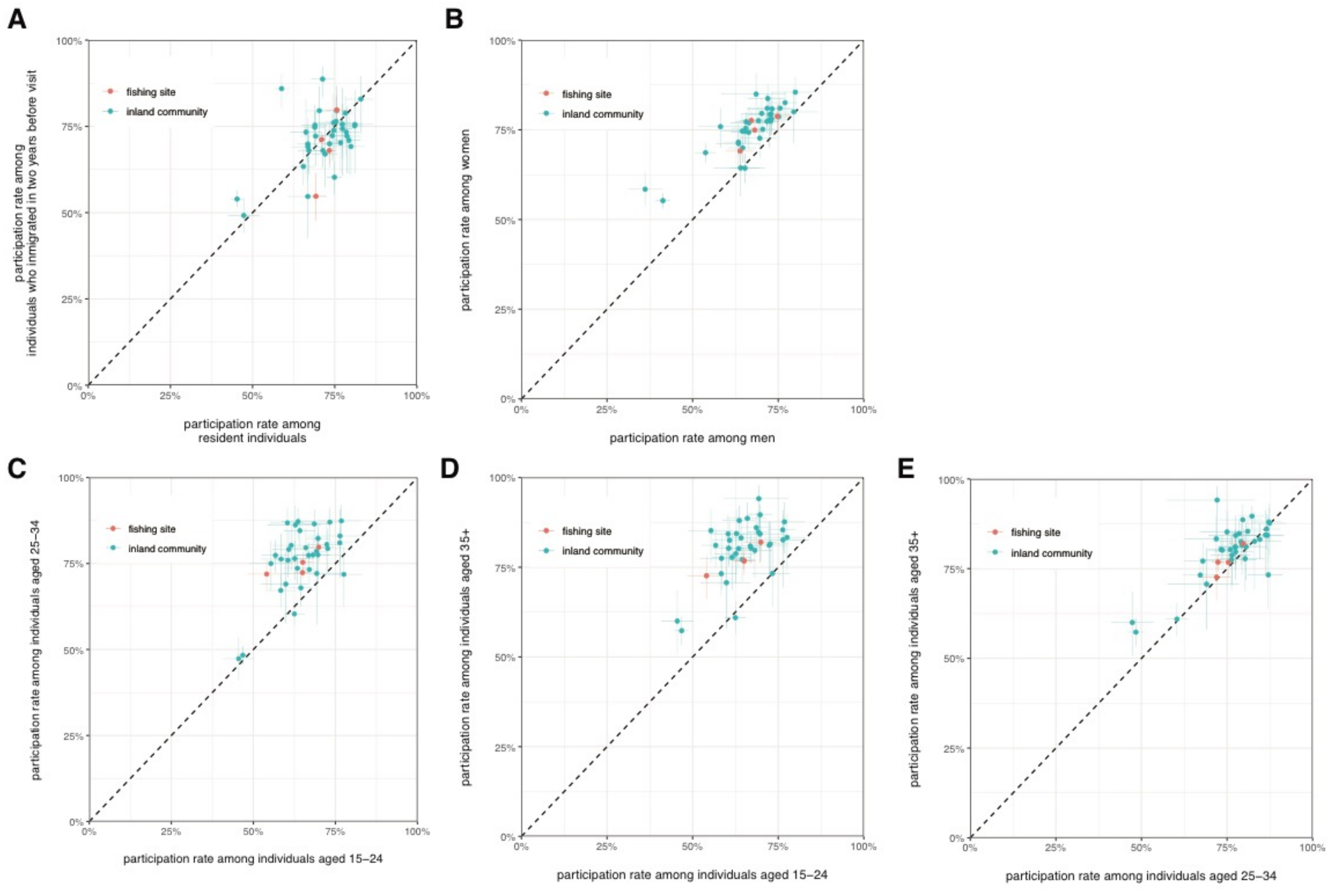

Figure S6. Participation rates by gender, age, and migration status. RCCS participation rates were defined as the number of participants divided by the number of census eligible individuals for given population strata in each RCCS community. 95% Agresti-Coull confidence intervals were calculated. The subfigures compare community-specific participation rates between two strata, (**A**) migration status, (**B**) gender, (**C**-**E**) age brackets. The diagonal line indicates no community-specific differences in participation rates for the two strata compared.

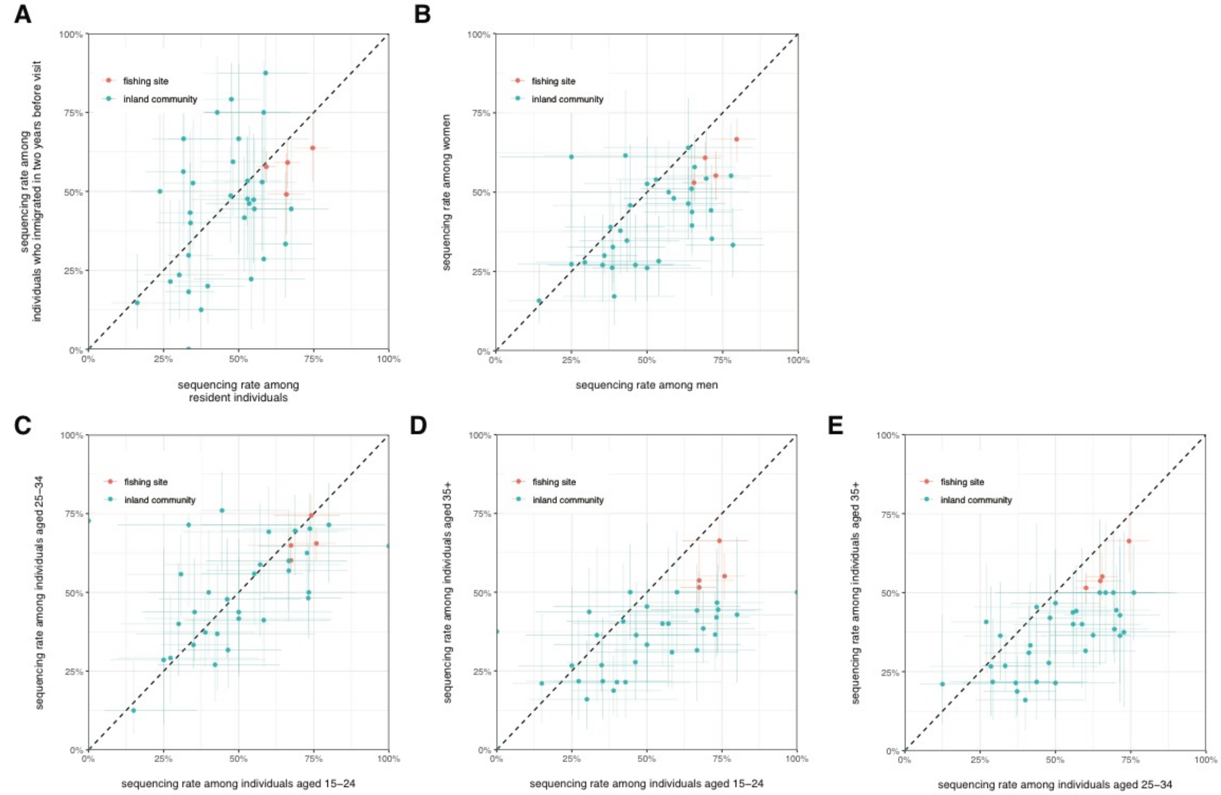

Figure S7. Sequence sampling rates by gender, age, and migration status. RCCS sequence sampling rates were defined as the number of individuals of whom virus was deep-sequenced at minimum quality criteria divided by the number of infected individuals who did not report ART use for given population strata in each RCCS community. 95% Agresti-Coull confidence intervals were calculated. The subfigures compare community-specific participation rates between two strata, (**A**) migration status, (**B**) gender, (**C**-**E**) age brackets. The diagonal line indicates no community-specific differences in sequence sampling rates for the two strata compared.

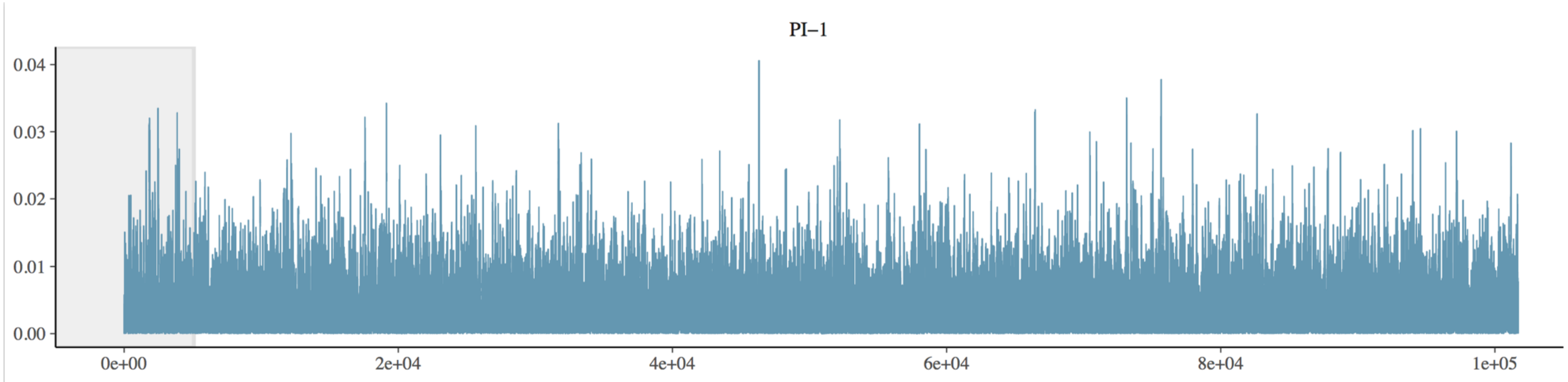

Figure S8. Numerical performance: traceplots for estimated proportions of transmission flows between RCCS communities. Parameter states for the first dimension of the target parameter $\boldsymbol{\pi}$ (proportions of transmission flows) from the first to the last full Markov Chain Monte Carlo cycle over all unknown parameters. The MCMC algorithm was run for 100,000 cycles, corresponding to 4·8 million MCMC iterations, of which the first 5% were discarded as burn-in. The traceplot indicates fast numerical convergence to the posterior distribution and good mixing on the posterior distribution.

Supplementary Text S3 Prediction of transmission flows between lakeside fishing and inland populations

Overview

The RCCS is not a proportionate sample of the underlying population in the Rakai region, chiefly because the RCCS oversamples Lake Victoria fishing communities. This means that estimated transmission flows between the communities within the cohort do not scale to the total population. We predicted the transmission flows $\boldsymbol{\pi}^* = (\pi^*_{FF}, \pi^*_{IF}, \pi^*_{FI}, \pi^*_{II})$ between the inland and fishing areas defined in Figure 1A. The predictions were based on the estimated transmission flows between RCCS communities, and scaled by the total population in inland and fishing areas.

Input data

The predictions required spatial estimates of the number of men and women in inland and fishing areas. High resolution estimates of population density on a 1km^2 spatial grid were from the World Pop Project, aggregated within inland and fishing areas by gender, and are reported in Supplementary Table S4 (7, 9). The population count in fishing areas was lower than the census-eligible population in the four fishing communities of the RCCS, suggesting underestimation of the population in the lakeside area by a factor of at least 1.78. We multiplied the World Pop estimate of the population size in the lakeside area by a factor of 2. Sensitivity analyses using alternative approaches did not substantially impact on our results as described in Supplementary Text S4. Population counts in the inland area agreed with estimates from the Ugandan Bureau of Statistics, and were left unchanged. For the predictions, we used the proportions ζ_I and ζ_F of individuals in inland and lakeside areas that are part of the RCCS survey.

Prediction of area-level transmission flows

Predictions were based on the posterior predictive distribution

$$p(\boldsymbol{\pi}^* \mid \boldsymbol{n}) = \int p(\boldsymbol{\pi}^* | \boldsymbol{\pi}, \boldsymbol{z}, Z, \boldsymbol{\xi}) \; p(\boldsymbol{\pi}, \boldsymbol{z}, Z, \boldsymbol{\xi} | \boldsymbol{n}) \; d\boldsymbol{\pi}, \boldsymbol{z}, Z, \boldsymbol{\xi}$$

where $p(\boldsymbol{\pi}, \boldsymbol{z}, Z, \boldsymbol{\xi} | \boldsymbol{n})$ is the joint posterior distribution of the parameters of the Rakai source attribution model in section S2.2, with strata collapsed to gender and area type. The vector of RCCS transmission flows was thus (inland:M -> inland:F, inland:M -> lakeside:F, inland:F -> inland:M, inland:F -> lakeside:M, lakeside:M -> lakeside:F, lakeside:M -> inland:F, lakeside:F -> lakeside:M, lakeside:F -> inland:M), of length $L^* = 8$. The estimated transmission flows between RCCS inland and fishing communities were then adjusted by the number of individuals under surveillance in the same manner as for sequence sampling in section S2.1, through the density

$$p(\boldsymbol{\pi}^* | \boldsymbol{\pi}, \boldsymbol{z}, Z, \boldsymbol{\xi}) = \int p(\boldsymbol{\pi}^*, \boldsymbol{z}^*, Z^*, \zeta | \boldsymbol{z}, Z) \; d\boldsymbol{\pi}^*, \boldsymbol{z}^*, Z^*, \zeta$$

where

$$p(\boldsymbol{\pi}^*, \boldsymbol{z}^*, Z^*, \zeta | \boldsymbol{\pi}, \boldsymbol{z}, Z) \propto \prod_{x,y} Binomial\big(z_{xy};\; z^*_{xy}, \zeta_x \zeta_y\big) \times Multinomial(\boldsymbol{z}^*;\; Z^*, \boldsymbol{\pi}^*) \times$$
$$p(\boldsymbol{\pi}^*) p(Z^*) p(\zeta).$$

The prior density for survey inclusion ζ_x in area x was set to the Beta distribution with parameters α_x set to the number of individuals surveyed in area x plus one, and β_x set to the number of individuals not surveyed in area x plus one. The prior density on the total number of transmission in inland and fishing areas, $p(Z^*)$, was set to a shifted Poisson distribution with mean $Z/\bar{\boldsymbol{\pi}}$. The prior density for the area-level transmission flows, $p(\boldsymbol{\pi}^*)$, was set to the Dirichlet distribution with parameters $0.8/L^*$.

Computational inference

Numerical estimation of the posterior predictive density of $\boldsymbol{\pi}^*$ was straightforward due to the low dimensionality of the parameter space ($L^* = 8$). First, 10,000 Monte Carlo samples were drawn from $p(\boldsymbol{\pi}, \boldsymbol{z}, Z, \boldsymbol{\xi} | \boldsymbol{n})$. Second, for each Monte Carlo sample, 100 samples from $p(\boldsymbol{\pi}^*, \boldsymbol{z}^*, Z^*, \zeta | \boldsymbol{\pi}_i, \boldsymbol{z}_i, Z_i)$ were generated using the same MCMC algorithm as in section S2.1. Numerical convergence was assessed with the Gelman-Rubin statistic, and was achieved in a

burn-in period of 90 iterations. Third, the 10 last MCMC iterations were retained, and merged across all 10,000 samples from $p(\boldsymbol{\pi}, \boldsymbol{z}, Z, \boldsymbol{\xi}|\boldsymbol{n})$. Figure S9 reports traceplots of $\boldsymbol{\pi}^*$, and traceplots for the other variables were similar. Effective sample sizes were calculated as described in Supplementary Text S2, and the smallest effective sample size was above 10,000, indicating good numerical performance.

Reported quantities

Analogous to the reported quantities described in section S2.2.

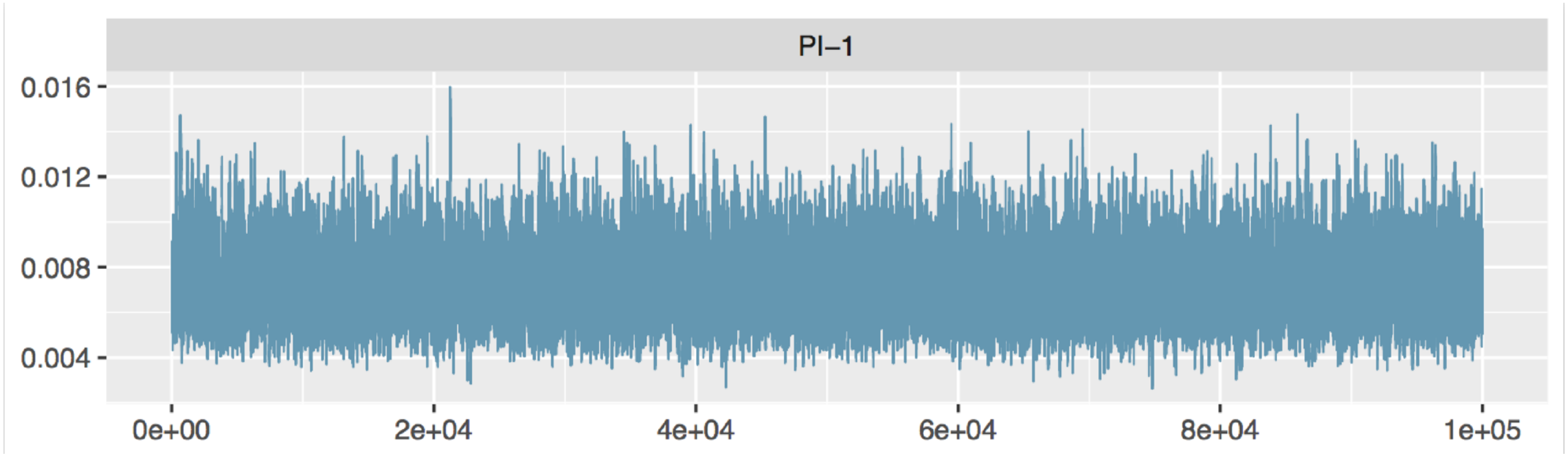

Figure S9. Numerical performance: traceplots for predicted proportions of transmission flows between inland and fishing sub-districts**.** Parameter states for the first component of $\boldsymbol{\pi}^*$ (predicted proportions of transmission flows). First, 10,000 Monte Carlo samples were drawn from $p(\boldsymbol{\pi}, \boldsymbol{z}, Z, \boldsymbol{\xi}|\boldsymbol{n})$ described in Supplementary Text S3. Second, for each Monte Carlo sample, 100 samples from $p(\boldsymbol{\pi}^*, \boldsymbol{z}^*, Z^*, \zeta|\boldsymbol{\pi}_i, \boldsymbol{z}_i, Z_i)$ were generated using the same MCMC algorithm as in Supplementary Text S2, section S2.1. Third, the 10 last MCMC iterations were retained in each case, and merged across all 10,000 samples from $p(\boldsymbol{\pi}, \boldsymbol{z}, Z, \boldsymbol{\xi}|\boldsymbol{n})$, to obtain 100,000 samples from $p(\boldsymbol{\pi}^*|\boldsymbol{n})$. The traceplot indicates good sampling of the posterior distribution.

Supplementary Text S4 Sensitivity Analyses

Impact of quality criteria on deep-sequence depth used to select virus from individuals for phylogenetic analysis

Deep-sequencing was attempted from viral samples of nearly all participant who self-reported to be ART naïve; however the quality of deep-sequencing output was moderate. For the main analysis, participants were included if they had virus deep-sequenced with viral sequence fragments of at least 250bp that covered the HIV genome at a depth of 30 copies (30X) for at least 750nt of the viral genome. In sensitivity analyses, these inclusion criteria were relaxed and tightened as follows:

Analysis	Description
10X	Include participants in phylogenetic analysis if they had virus deep-sequenced with viral sequence fragments of at least 250bp that covered the HIV genome at a depth of 10 copies (10X) for at least 750nt of the viral genome.
20X	Include participants in phylogenetic analysis if they had virus deep-sequenced with viral sequence fragments of at least 250bp that covered the HIV genome at a depth of 20 copies (20X) for at least 750nt of the viral genome.
30X (central)	Include participants in phylogenetic analysis if they had virus deep-sequenced with viral sequence fragments of at least 250bp that covered the HIV genome at a depth of 30 copies (30X) for at least 750nt of the viral genome.
50X	Include participants in phylogenetic analysis if they had virus deep-sequenced with viral sequence fragments of at least 250bp that covered the HIV genome at a depth of 50 copies (50X) for at least 750nt of the viral genome.

Figure S10 shows the impact of these criteria on estimated transmission flows among RCCS communities by gender. Figure S11 illustrates the impact of these criteria on the transmission flow ratio from inland to fishing communities rather than vice versa overall. The interquartile range of the estimated transmission flow ratio was clearly >1 at stronger quality criteria (20X to 50X), but not in the 10X analysis.

Impact of stringency criteria on the proportion of deep-sequence phylogenies that are supporting linkage and transmission in one particular direction

Using phyloscanner, many deep-sequence phylogenies were reconstructed for each pair of individuals, and phylogenetic inferences are based on the frequency of phylogenetic relationships seen in this set of deep-sequence phylogenies. For the main analysis, a pair of sequenced participants was classified as a source-recipient pair and used for estimating transmission flows if at least 60% of deep-sequence phylogenies supported virus transmission, and if at least 60% of these phylogenies supported one direction of transmission. The error rate in phylogenetic inference of the direction of transmission based on this criterion was estimated to be within 10-20%. In sensitivity analyses, the threshold was varied as follows:

Analysis	Description
50%	A pair of sequenced participants was classified as a source-recipient pair and used for estimating transmission flows if at least 50% of deep-sequence phylogenies supported virus transmission, and if at least 50% of these phylogenies supported one direction of transmission.
55%	A pair of sequenced participants was classified as a source-recipient pair and used for estimating transmission flows if at least 55% of deep-sequence phylogenies supported virus transmission, and if at least 55% of these phylogenies supported one direction of transmission.
60% (central)	A pair of sequenced participants was classified as a source-recipient pair and used for estimating transmission flows if at least 60% of deep-sequence phylogenies supported virus transmission, and if at least 60% of these phylogenies supported one direction of transmission.
65%	A pair of sequenced participants was classified as a source-recipient pair and used for estimating transmission flows if at least 65% of deep-sequence phylogenies supported virus transmission, and if at least 65% of these phylogenies supported one direction of transmission.
70%	A pair of sequenced participants was classified as a source-recipient pair and used for estimating transmission flows if at least 70% of deep-sequence phylogenies supported virus transmission, and if at least 70% of these phylogenies supported one direction of transmission.

Figure S12 shows the impact of these criteria on estimated transmission flows among RCCS communities by gender. Figure S13 illustrates the impact of these criteria on the transmission flow ratio from inland to fishing communities rather than vice versa overall. The width of the 95% credibility intervals increased as selection criteria were stricter, and the interquartile range of the estimated transmission flow ratio was clearly >1 in all cases.

Impact of classification of phylogenetically likely transmitters into residents and in-migrants
To interpret phylogenetically reconstructed source-recipient pairs, we used data on current residence (geo-location of current household) and in-migration (date and origin of in-migration). The geo-location of each phylogenetically likely recipient partner was set to the community in which the recipient was found to be infected. For the main analysis, the location of the phylogenetically likely transmitter was set to the community of residence at or shortly before the recipient was identified as HIV-positive. If the source partner had migrated within the two prior years, the location was set as the community prior to migration. In sensitivity analyses, the timespan used to classify the source partner as a recent in-migrant was varied as follows:

Analysis	Description
6 months	If the source partner had migrated within the 6 prior months, the location was set as the community prior to migration.
12 months	If the source partner had migrated within the 12 prior months, the location was set as the community prior to migration.
24 months (central)	If the source partner had migrated within the 24 prior months, the location was set as the community prior to migration.
36 months	If the source partner had migrated within the 36 prior months, the location was set as the community prior to migration.
48 months	If the source partner had migrated within the 48 prior months, the location was set as the community prior to migration.

Figure S14 shows the impact of these criteria on estimated transmission flows among RCCS communities by gender. Figure S15 illustrates the impact of these criteria on the transmission flow ratio from inland to fishing communities rather than vice versa overall. The interquartile range of the estimated transmission flow ratio was clearly >1 in all sensitivity analyses.

Impact of unknown origins of migration
There were 5 phylogenetically likely transmitters who had in-migrated in the two years prior to diagnosis of the likely recipient, and for whom the origin of migration could not be identified. For the main analysis, the source location was set to fishing communities in order to obtain a conservative estimate of transmission flows that is biased towards transmissions from fishing communities. In a sensitivity analysis, the source location of these likely transmitters was set to inland communities:

Analysis	Description
Inland communities	Source location of 5 phylogenetically likely transmitters with unknown origin of migration was set to inland communities.
Fishing communities (central)	Source location of 5 phylogenetically likely transmitters with unknown origin of migration was set to fishing communities.

Figure S16 shows the impact of these criteria on estimated transmission flows among RCCS communities by gender. Figure S17 illustrates the impact of these criteria on the transmission flow ratio from inland to fishing communities rather than vice versa overall. The interquartile range of the estimated overall transmission flow ratio was clearly >1 in all sensitivity analyses.

Impact of sampling adjustments
Viral phylogenetic estimates of transmission flows are derived from reconstructed viral phylogenies, which in turn depend on who is sampled. For the main analysis, we adjusted crude estimates by variation in the proportion of census-eligible individuals who participated, by variation in the proportion of infected participants not reporting ART use at first visit who were deep-sequenced at minimum quality criteria, and by variation in the proportion of

infected participants who were deep-sequenced at minimum quality criteria. In sensitivity analyses, we varied these adjustments as follows:

Analysis	Description
P:0, S:0	No adjustments for variation in participation probability, and no adjustments for variation in deep-sequencing probability.
P:1, S:0	With adjustments for variation in participation probability, and no adjustments for variation in deep-sequencing probability.
P:0, S:1	No adjustments for variation in participation probability, and with adjustments for variation in the proportion of sequenced individuals among individuals who did not report ART use at their first visit.
P:1, S:1 (central)	With adjustments for variation in participation probability, and with adjustments for variation in the proportion of sequenced individuals among individuals who did not report ART use at their first visit.
P:0, S:2	No adjustments for variation in participation probability, and with adjustments for variation in the proportion of sequenced individuals among infected individuals including those reporting ART use at their first visit.
P:1, S:2	With adjustments for variation in participation probability, and with adjustments for variation in the proportion of sequenced individuals among infected individuals including those reporting ART use at their first visit.

Figure S18 shows the impact of these criteria on estimated transmission flows among RCCS communities by gender. Figure S19 illustrates the impact of these criteria on the transmission flow ratio from inland to fishing communities rather than vice versa overall. The interquartile range of the estimated transmission flow ratio was clearly >1 in all sensitivity analyses.

Impact of population size estimates

The statistical predictions of transmission flows between the lakeside and inland areas shown in Figure 1A were based on the estimated transmission flows among RCCS communities and population size data on the lakeside and inland areas (7, 9). For the main analysis, we used the 2015 WorldPop estimate of the total number of individuals living in inland and fishing areas by gender reported in Supplementary Table S4, multiplied counts due to underestimation by a factor of 2, and the divided the number of census-eligible individuals by these counts to obtain estimates of the proportion of survey-eligible individuals in lakeside and fishing areas. This proportion was then used to scale transmission flows within and between RCCS communities to lakeside and inland areas as described in Supplementary Text S3. In sensitivity analyses, we varied the underestimation factor from 1 to 3. In addition, we used estimates of the infected population in lakeside and inland areas to scale transmission flows, using a spatial mapping approach that we previously reported (7). Briefly, spatial maps of infected men and women aged 15-49 years were generated on a 1km^2 high-resolution grid through a spatial binomial-logistic disease count model in Stan. The estimated maps included data on (1) population density from the World Pop Project, (2) age structure from the RCCS census conducted in 2015-2016, and (3) geo-referenced HIV prevalence data (15-49 years old) from the RCCS shown in Supplementary Figure S1. The spatial estimates of infected men and women were then aggregated within inland and fishing areas by gender, and are reported in Supplementary Table S4. The full set of sensitivity analyses was as follows:

Analysis	Description
Prop all, 1X	Scaling RCCS transmission flows by 1 times the WorldPop estimate of the total population size of men and women in inland and fishing areas.
Prop all, 2X	Scaling RCCS transmission flows by 2 times the WorldPop estimate of the total population size of men and women in inland and fishing areas.
Prop all, 3X	Scaling RCCS transmission flows by 3 times the WorldPop estimate of the total population size of men and women in inland and fishing areas.
Prop infected, 1X	Scaling RCCS transmission flows by 1 times the estimate of the number of HIV-infected men and women in inland and fishing areas.
Prop infected, 2X	Scaling RCCS transmission flows by 2 times the estimate of the number of HIV-infected men and women in inland and fishing areas.

Prop infected, 3X	Scaling RCCS transmission flows by 3 times the estimate of the number of HIV-infected men and women in inland and fishing areas.

Figure S20 shows the impact of these criteria on estimated transmission flows among RCCS communities by gender. Figure S21 illustrates the impact of these criteria on the transmission flow ratio from inland to fishing communities rather than vice versa overall. The interquartile range of the estimated transmission flow ratio was clearly >1 in all sensitivity analyses.

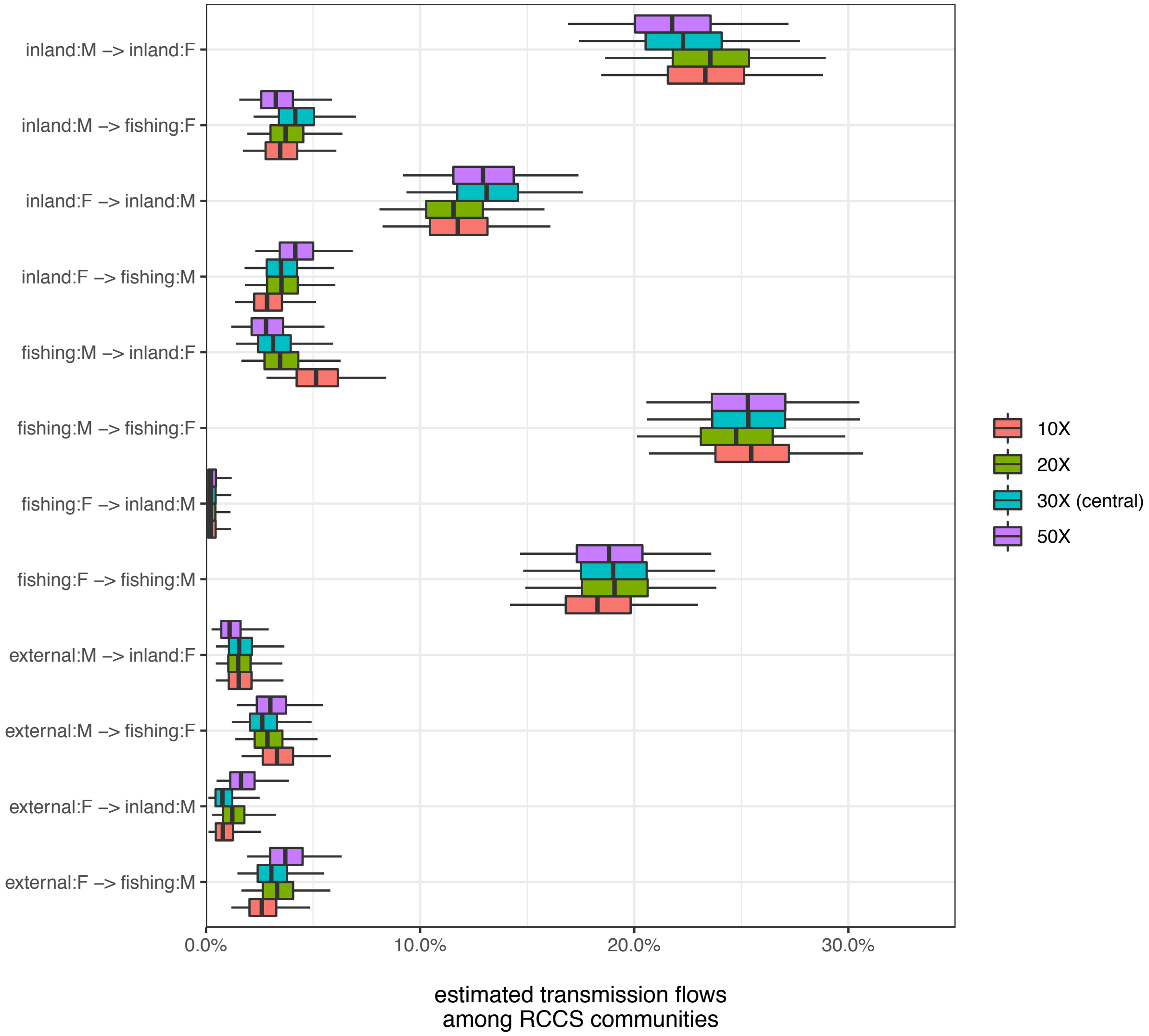

Figure S10. Impact of quality criteria on deep-sequencing depth on estimated transmission flows.

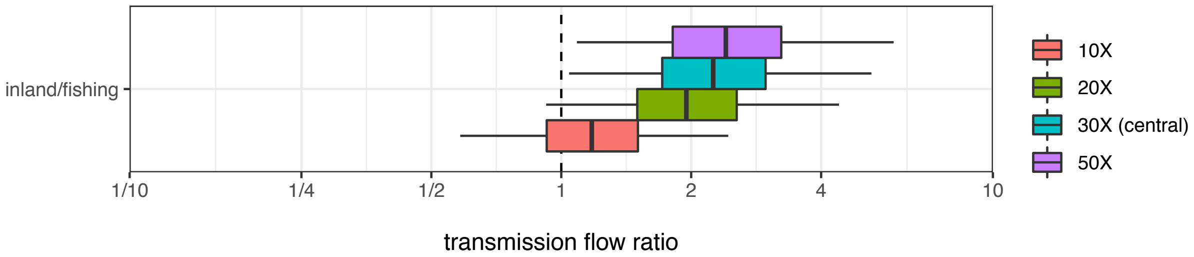

Figure S11. Impact of quality criteria on deep-sequencing depth on the estimated transmission flow ratio from inland communities to fishing communities rather than vice versa.

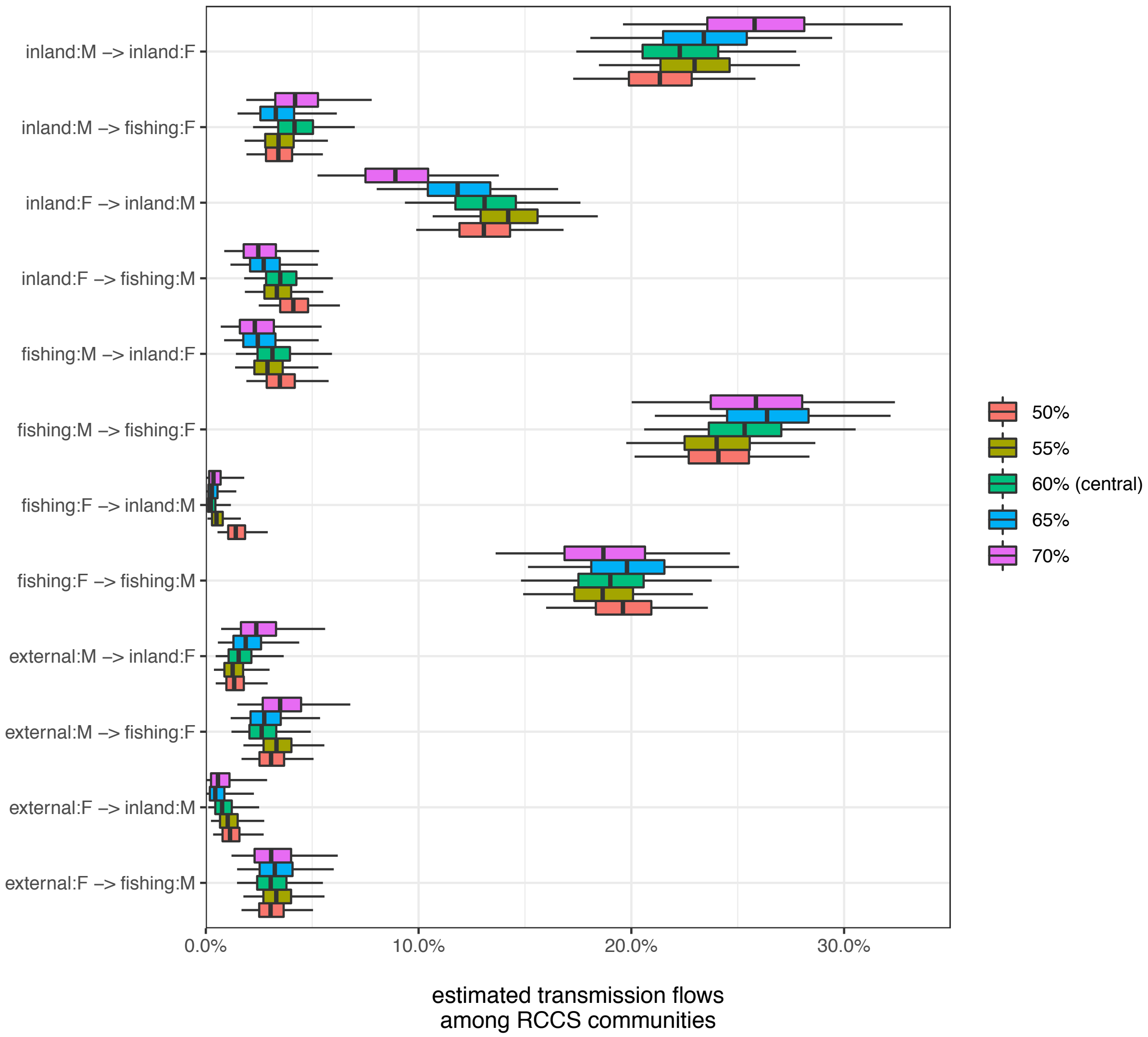

Figure S12. Impact of stringency criteria on the proportion of deep-sequence phylogenies in support of transmission and direction of transmission by gender on estimated transmission flows.

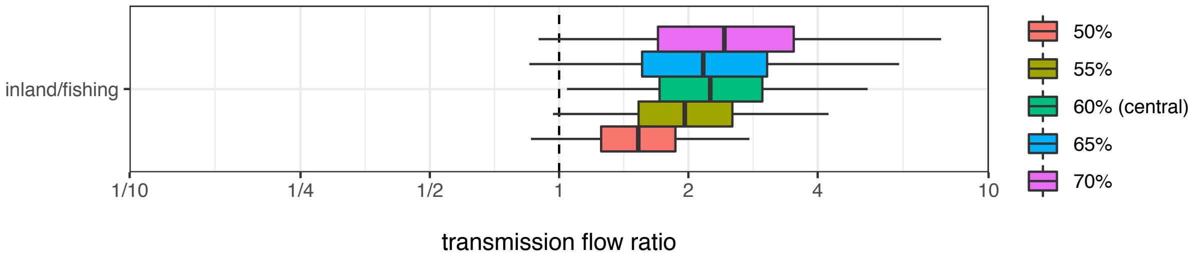

Figure S13. Impact of stringency criteria on the proportion of deep-sequence phylogenies in support of transmission and direction of transmission on the estimated transmission flow ratio from inland communities to fishing communities rather than vice versa.

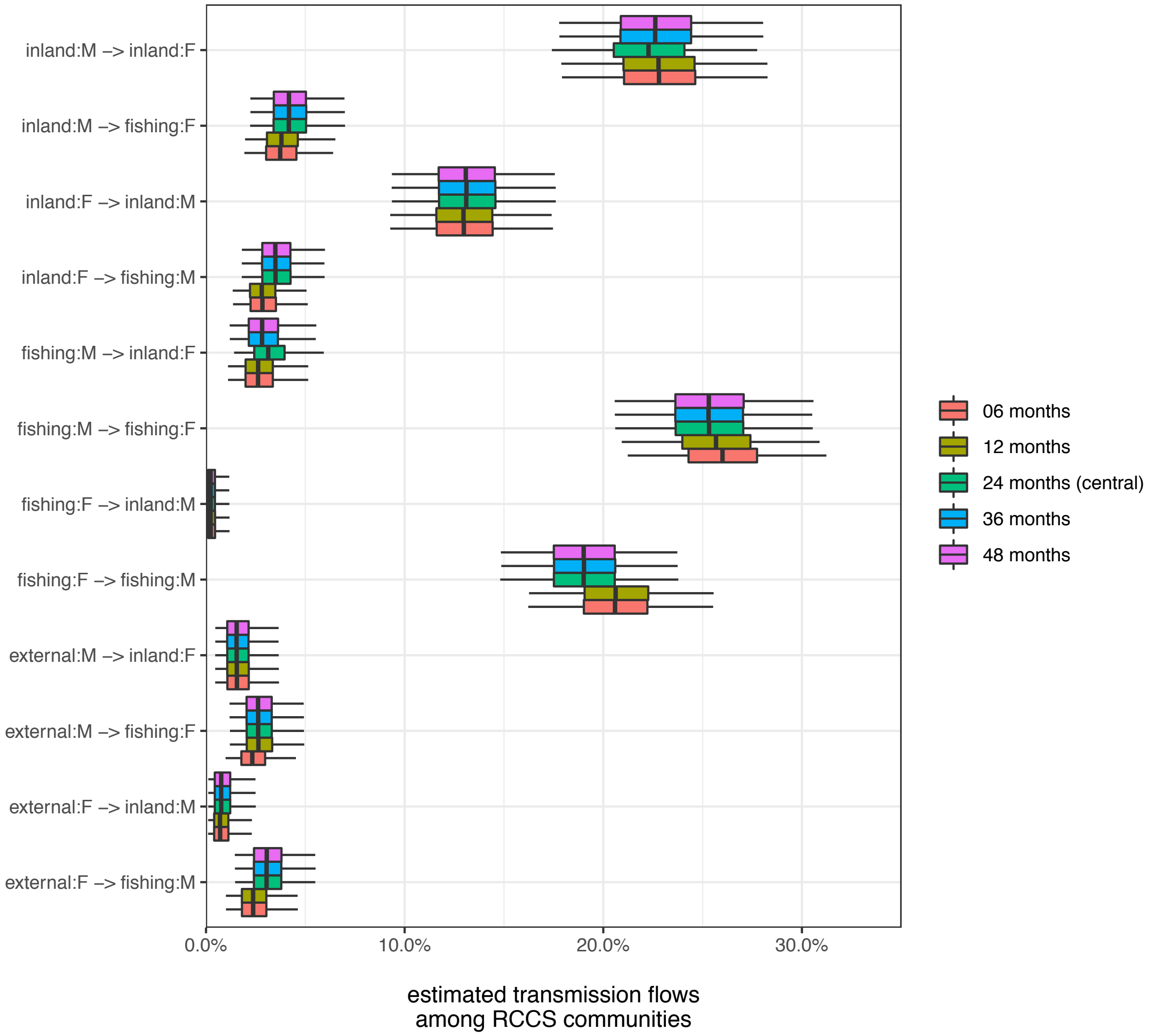

Figure S14. Impact of the time span between in-migration events of likely transmitters and time of diagnosis of likely recipients within which the source location is set to the origin of migration on estimated transmission flows by gender.

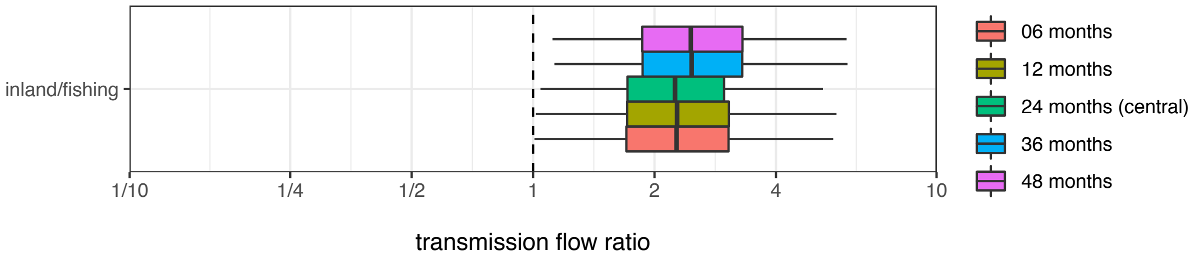

Figure S14. Impact of the time span between in-migration events of likely transmitters and time of diagnosis of likely recipients within which the source location is set to the origin of migration on the estimated transmission flow ratio from inland communities to fishing communities rather than vice versa.

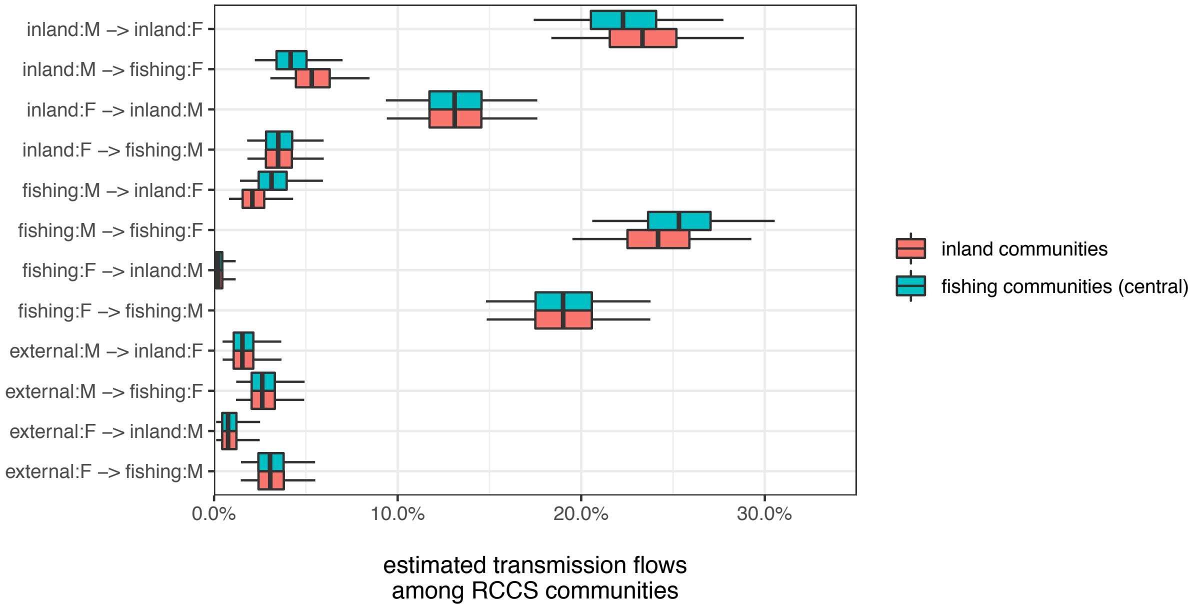

Figure S16. Impact of attributing the source location of likely transmitters with unknown origin of migration to inland or fishing communities on estimated transmission flows by gender.

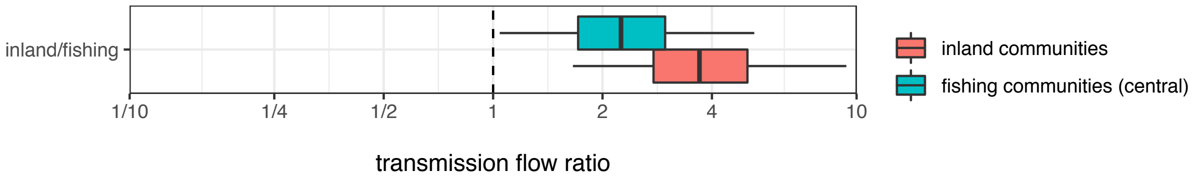

Figure S17. Impact of attributing the source location of likely transmitters with unknown origin of migration to inland or fishing communities on the estimated transmission flow ratio from inland communities to fishing communities rather than vice versa.

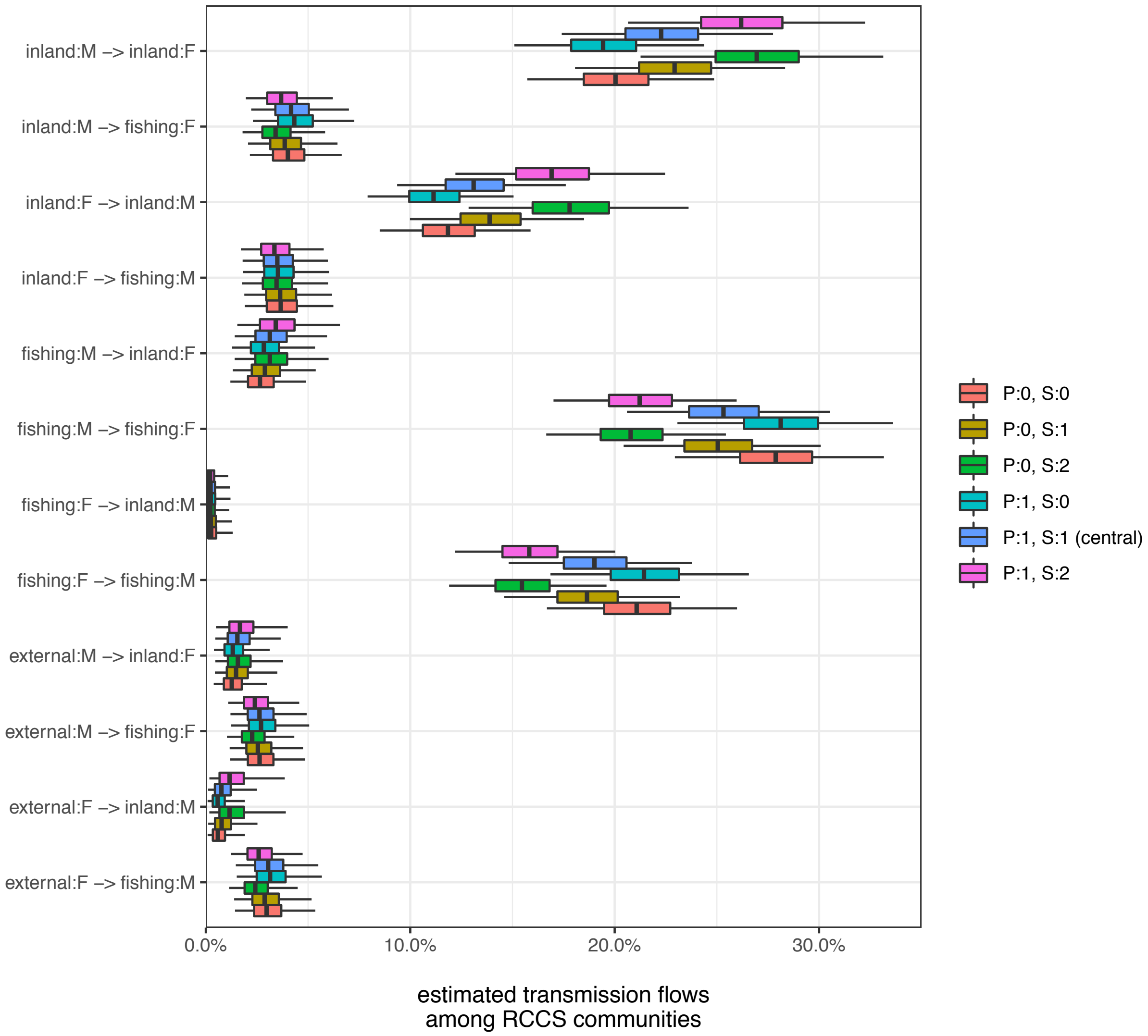

Figure S18. Impact of adjustments for variation in participation rates and for variation in deep-sequencing rates of population groups defined by gender, age, migration status, and RCCS community on estimated transmission flows by gender.

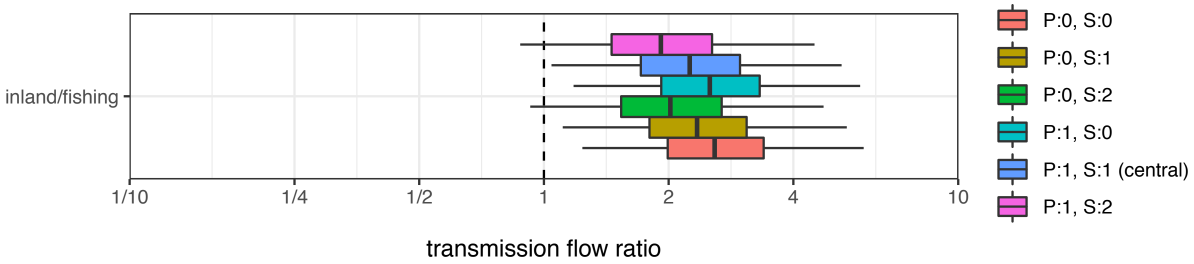

Figure S19. Impact of adjustments for variation in participation rates and for variation in deep-sequencing rates of population groups defined by gender, age, migration status, and RCCS community on the estimated transmission flow ratio from inland communities to fishing communities rather than vice versa.

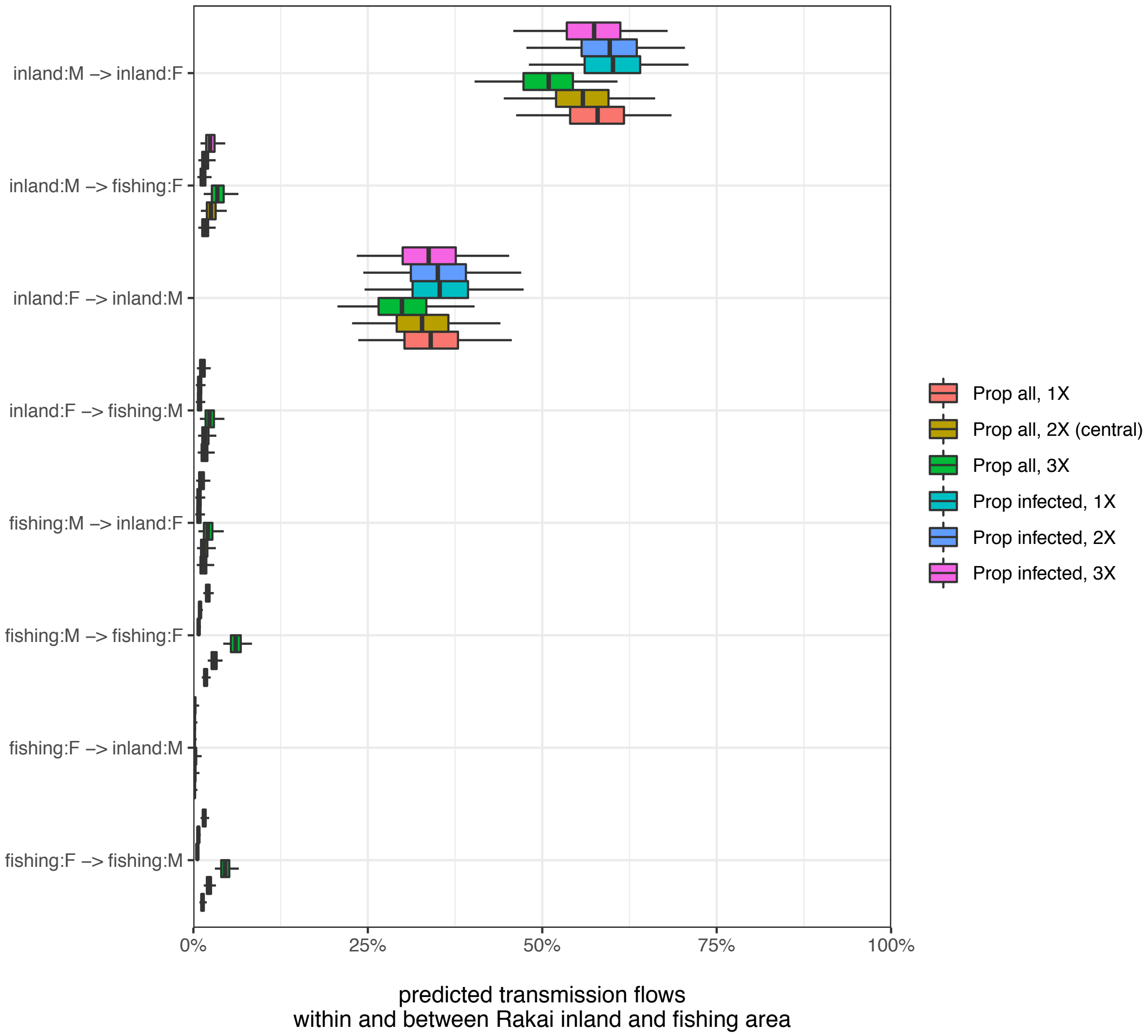

Figure S20. Impact of population size estimates on predicted transmission flows in inland and fishing areas by gender.

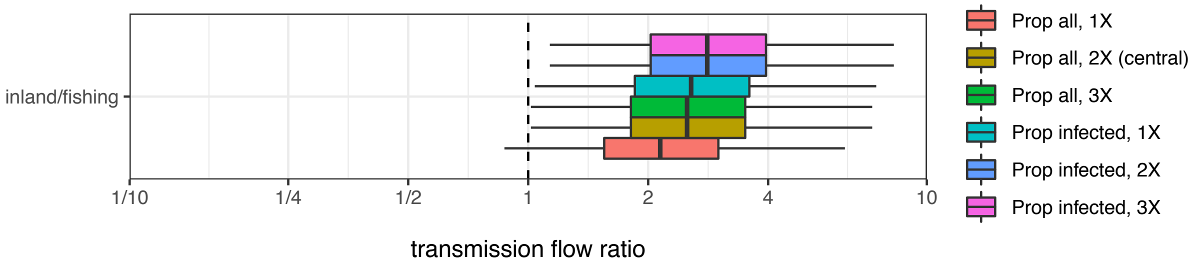

Figure S21. Impact of population size estimates on the predicted transmission flow ratio from inland to lakeside area rather than vice versa.

Bibliography

1. Grabowski MK, Reynolds SJ, Kagaayi J, Gray RH, Clarke W, Chang LW, et al. The validity of self-reported antiretroviral use in persons living with HIV: a population-based study. AIDS. 2018;32(3):363-9.
2. Grabowski MK, Serwadda DM, Gray RH, Nakigozi G, Kigozi G, Kagaayi J, et al. HIV Prevention Efforts and Incidence of HIV in Uganda. N Engl J Med. 2017;377(22):2154-66.

3. Ratmann O, Wymant C, Colijn C, Danaviah S, Essex M, Frost SDW, et al. HIV-1 full-genome phylogenetics of generalized epidemics in sub-Saharan Africa: impact of missing nucleotide characters in next-generation sequences. AIDS Res Hum Retroviruses. 2017;33(11):1083-98.
4. Ratmann O, Grabowski MK, Hall M, Golubchik T, Wymant C, Abeler-Dorner L, et al. Inferring HIV-1 transmission networks and sources of epidemic spread in Africa with deep-sequence phylogenetic analysis. Nat Commun. 2019;10(1):1411.
5. Wymant C, Hall M, Ratmann O, Bonsall D, Golubchik T, de Cesare M, et al. PHYLOSCANNER: Inferring Transmission from Within- and Between-Host Pathogen Genetic Diversity. Mol Biol Evol. 2017.
6. Kagaayi J, Chang LW, Ssempijja V, Grabowski MK, Ssekubugu R, Nakigozi G, et al. Impact of combination HIV interventions on HIV incidence in hyperendemic fishing communities in Uganda: a prospective cohort study. Lancet HIV. 2019;6(10):e680-e7.
7. Chang LW, Grabowski MK, Ssekubugu R, Nalugoda F, Kigozi G, Nantume B, et al. Heterogeneity of the HIV epidemic in agrarian, trading, and fishing communities in Rakai, Uganda: an observational epidemiological study. Lancet HIV. 2016;3(8):e388-e96.
8. Carpenter B, Gelman A, Hoffman MD, Lee D, Goodrich B, Betancourt M, et al. Stan: A probabilistic programming language. Journal of Statistical Software 2017;76(1).
9. Tatem AJ. WorldPop, open data for spatial demography. Sci Data. 2017;4:170004.
